# Efgartigimod Versus Lymphoplasmapheresis as Preoperative Rapid Antibody‐Clearing Therapies for Thymectomy in Generalized Myasthenia Gravis: Effectiveness, Safety and Cost Outcomes Compared to Conventional Preparation

**DOI:** 10.1002/cns.70993

**Published:** 2026-06-19

**Authors:** Qian Zhou, Ting He, Yuanda Cheng, Kangzhi Chen, Yuzhen Ouyang, Xiaohua Dong, Guanzhong Shi, Zeyi Wen, Huan Yang

**Affiliations:** ^1^ Department of Neurology, Xiangya Hospital Central South University Changsha Hunan People's Republic of China; ^2^ Clinical Research Center for Neuroimmune and Neuromuscular Disorders, Xiangya Hospital Central South University Changsha Hunan People's Republic of China; ^3^ National Clinical Research Center for Geriatric Disorders, Xiangya Hospital Central South University Changsha Hunan People's Republic of China; ^4^ Department of Thoracic Surgery, Xiangya Hospital, National Clinical Research Center for Geriatric Disorders Central South University Changsha People's Republic of China; ^5^ Department of Neurology and Immunobiology Yale School of Medicine New Haven Connecticut USA

**Keywords:** effectiveness, efgartigimod, generalized myasthenia gravis, lymphoplasmapheresis, POMC, thymectomy

## Abstract

**Aims:**

To compare the real‐world effectiveness, surgical and economic outcomes of preoperative rapid antibody clearance therapy (RACT: lymphoplasmapheresis (LPE), efgartigimod (EFG)) versus oral immunosuppressants (Oral IS) in generalized myasthenia gravis (gMG) patients undergoing thymectomy.

**Methods:**

This retrospective study included 78 patients (36 Oral IS, 42 RACT: 25 LPE, 17 EFG). Primary outcome: 3‐month postoperative quantitative myasthenia gravis (QMG) score. Secondary outcomes: 1‐month postoperative exacerbation rate, 3‐ and 6‐month MG activities of daily living (MG‐ADL), 6‐month QMG, surgical outcomes (operative time, blood loss, postoperative myasthenic crisis (POMC)), perioperative costs (pre‐ and post‐reimbursement). Propensity score overlap weighting balanced covariates. Sensitivity analyses used propensity matching; multiple imputation handled missing data.

**Results:**

After weighting, 3‐month QMG scores showed no significant difference between RACT and Oral IS groups (mean difference (MD) −0.23, 95% CI: −2.99 to 2.54, *p* = 0.831) nor between the EFG and LPE subgroups (MD 1.15, 95% CI: −3.97 to 6.26, *p* = 0.567); similar for MG‐ADL. All groups improved over time (*p* < 0.001). For EFG vs. LPE, 1‐month exacerbation rate was 43.3% vs. 26.1% (*p* = 0.412). No significant between‐group differences between EFG and LPE were found in 6‐month QMG/MG‐ADL scores and surgical outcomes. EFG had higher preoperative pre‐reimbursement costs (MD 14,500 CNY, *p* = 0.026), but no cost differences remained after insurance. Sensitivity analyses confirmed robustness of the primary findings.

**Conclusion:**

Preoperative RACT effectively reduced QMG in gMG patients, with no significant postoperative medium‐term effectiveness and safety differences between RACT and Oral IS. Similarly, no significant differences in postoperative medium‐term effectiveness, safety, or post‐reimbursement costs were observed between EFG and LPE.

## Graphical abstract

In this exploratory study, no statistically significant differences in effectiveness, safety, or cost were observed between EFG and LPE, nor between RACT and oral immunosuppressants for pre‐thymectomy preparation in MG. These findings are hypothesis‐generating and warrant prospective validation.

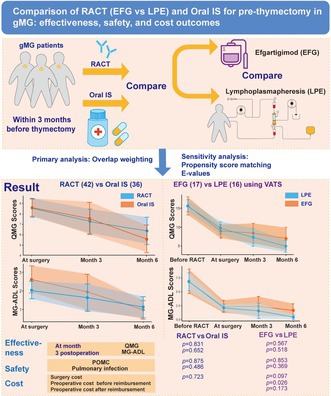

**Abbreviations**
Ab: antibody
AChR‐Ab: acetylcholine receptor antibodies
AIC: Akaike Information Criterion
AZA: Azathioprine
BMI: body mass index
CI: confidence intervals
COPD: chronic obstructive pulmonary disease
EFG: Efgartigimod
GEE: Generalized estimating equations
IQR: interquartile range
IS: immunosuppressants
IVIG: intravenous immunoglobulin
LPE: lymphoplasmapheresis
MG: myasthenia gravis
MG‐ADL: MG Activities of Daily Living
MGFA‐CC: Myasthenia Gravis Foundation of America Clinical Classification
MMF: mycophenolate mofetil
OPEN: open sternotomy
PE: plasma exchange
POMC: postoperative myasthenic crises
Pred: prednisone
QMG: quantitative myasthenia gravis
RACT: rapid antibody clearance therapy
RATS: robot‐Assisted Thoracic surgery
ROC: receiver operating characteristic
RyR: Ryanodine Receptor
SD: standard deviation
SMD: standardized mean differences
TAC: tacrolimus
TAMG: thymoma‐associated MG
VATS: video‐assisted thoracoscopic surgery
VIF: variance inflation factors

## 1 Introduction

Myasthenia gravis (MG) is an antibody‐mediated autoimmune disorder characterized by fluctuating muscle weakness [1]. Approximately 80%–85% of MG patients test positive for acetylcholine receptor antibodies (AChR‐Ab) [2], and the disease is often associated with thymic abnormalities like thymoma or thymic hyperplasia, which contribute to its pathogenesis and progression [3]. Thymectomy is an effective intervention that improves clinical outcomes, enhances symptom control, and reduces the need for immunosuppressants [4]. Although considered elective in guidelines, early thymectomy is frequently recommended, especially in early‐onset generalized MG (gMG) [5].

Conventional immunosuppressive therapies often require months to take effect, and 15% of patients do not respond [6, 7]. Thymectomy carries perioperative risks including respiratory complications and postoperative myasthenic crisis (POMC), with severe or unstable preoperative symptoms being key predictors of poor outcome [8]. These findings underscore the importance of stabilizing disease activity before thymectomy, while rapid antibody clearance therapies (RACT) hold promise to achieve this, reducing quantitative myasthenia gravis (QMG) scores and shortening the preparation period [9]. However, it remains unclear whether these approaches, compared with oral immunosuppressants (Oral IS) alone, affect postoperative risks or long‐term outcomes.

Plasma exchange (PE) serves as a rescue therapy for acute exacerbation and preoperative optimization, potentially lowering POMC risk [10, 11], but its clinical utility is limited by cost, plasma availability, and adverse events [12]. An evolution of this technique, lymphoplasmapheresis (LPE), integrates lymphocyte apheresis with conventional PE, offering greater efficacy, shorter treatment time, and less plasma consumption [13]. It has been applied in preoperative MG patients [14], yet it remains costly and plasma‐dependent.

Efgartigimod (EFG), the first neonatal Fc receptor (FcRn) antagonist approved for gMG, works by blocking FcRn to rapidly reduce pathogenic immunoglobulin G (IgG) levels [15]. While effective in general management [16], its role in thymectomy preparation is less defined. A recent trial in thymoma‐associated MG (TAMG) patients showed that perioperative EFG rapidly lowered MG Activities of Daily Living (MG‐ADL) scores, facilitated surgery, and led to low POMC rates with a favorable safety profile [17]. Nonetheless, real‐world evidence is needed to confirm these benefits. Crucially, no studies have directly compared EFG with LPE in terms of effectiveness, safety, or cost‐effectiveness [18].

Therefore, this study aimed to compare effectiveness, safety, and cost outcomes in perioperative gMG patients prepared with LPE versus EFG, and to compare both groups against a control cohort on Oral IS with comparable preoperative QMG scores.

## 2 Methods

### 2.1 Study Population

This single‐center retrospective cohort study included 165 MG patients who underwent standard extended thymic/thymoma resection at the Department of Thoracic Surgery, Xiangya Hospital between January 2017 and May 2025, following the Declaration of Helsinki and approved by the Ethics Committee of Xiangya Hospital (approval number: 2025112118). Due to the retrospective design and de‐identified analysis, informed consent was waived.

**FIGURE 1:**
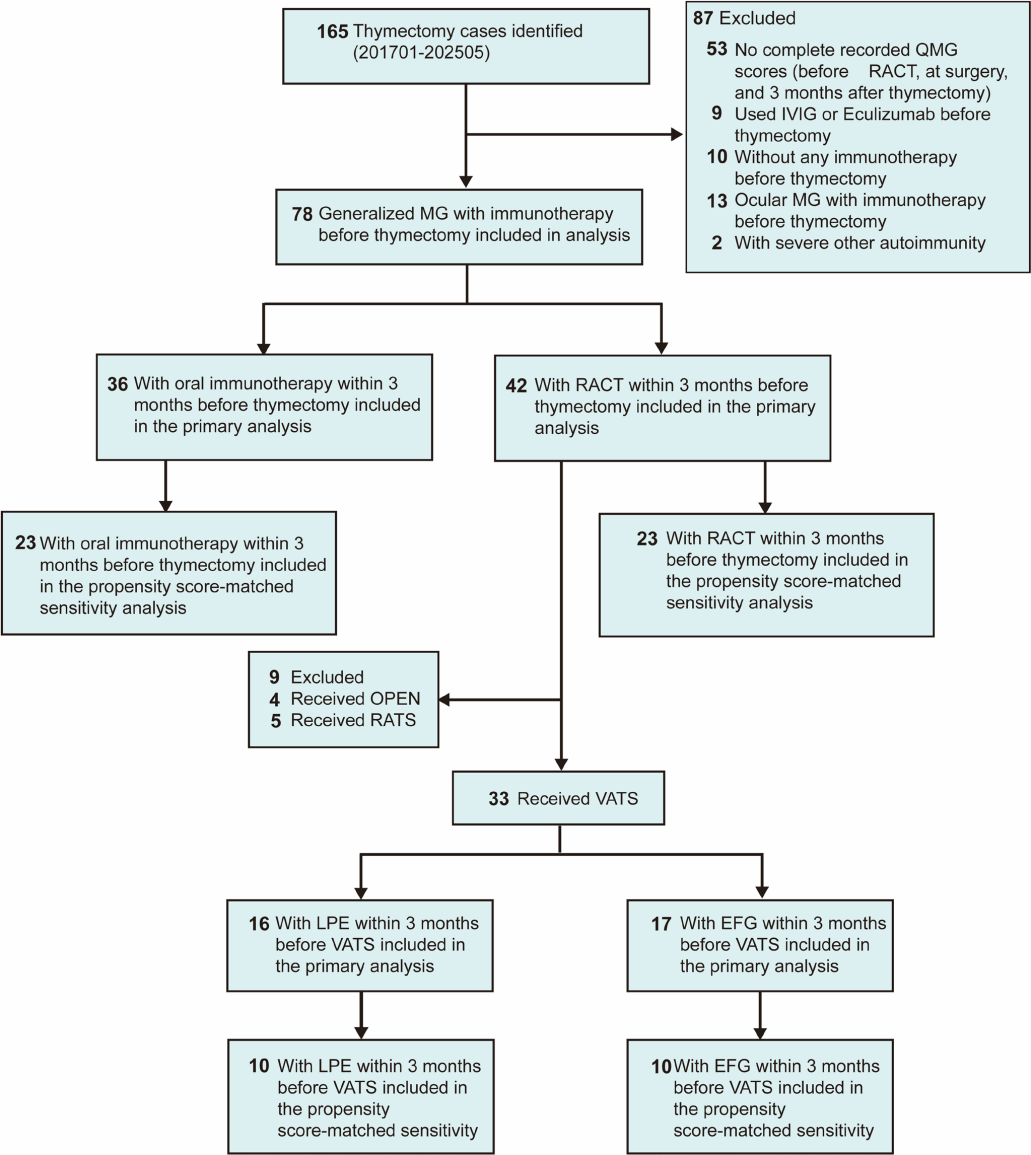
Identification of eligible patients and development of cohorts in the study.

GMG was diagnosed based on clinical manifestations including fluctuating muscle weakness affecting swallowing, speech, respiration, phonation, or limb/neck movements, and at least one positive result from the AChR‐Ab testing, repetitive nerve stimulation, or neostigmine challenge. Exclusion criteria included: (1) lost to follow‐up during the observation period; (2) use of intravenous immunoglobulin (IVIG) or Eculizumab within 3 months prior to thymectomy; (3) no preoperative immunotherapy (including Oral IS) or RACT (defined as only LPE or EFG); (4) ocular MG before thymectomy; or (5) coexisting severe autoimmune diseases that could confound MG assessment or treatment evaluation (Figure 1).

### 2.2 Baseline Characteristics and Potential Predictive Variables

Baseline data from the Xiangya Hospital MG database and electronic medical records included: (1) Demographics: age at onset, sex, and body mass index (BMI). (2) Clinical features: MG duration, antibody status, history of MG crisis (defined as intubation with/without mechanical ventilation), and prior immunosuppressive therapy use (≥ 1 month). QMG scores, MG‐ADL scores, and Myasthenia Gravis Foundation of America Clinical Classification (MGFA‐CC) were recorded at key time points: before RACT, at surgery, and at 3 and 6 months postoperatively. MGFA‐CC 0 indicated asymptomatic status at evaluation [19]. (3) Thymus pathology. (4) Comorbidities (e.g., autoimmune diseases, hypertension, diabetes), and chronic respiratory disorders (e.g., diffuse panbronchiolitis, bronchiectasis, chronic obstructive pulmonary disease (COPD)). (5) Surgical details: surgical approach (open sternotomy (OPEN), video‐assisted thoracoscopic surgery (VATS), or robot‐Assisted Thoracic surgery (RATS)), operation duration, blood loss, intensive care unit (ICU) length of stay, duration of assisted respiration, and pulmonary infection. (6) Financial burden: perioperative costs from 3 months before to 3 months after thymectomy, divided into preoperative, surgical, and postoperative phases.

### 2.3 Procedure for LPE and EFG

LPE was performed exclusively at Xiangya Hospital according to previously reported practices [14, 20]. Patients typically underwent 1 to 3 LPE procedures before thymectomy, tailored to their clinical condition, with a 3‐day interval between sessions. In the EFG group, patients received intravenous EFG at 10 mg/kg once weekly, with the number of doses adjusted based on individual therapeutic response.

### 2.4 Outcome Measures

The primary outcome was the QMG scores at 3 months post‐thymectomy. Secondary outcomes encompassed other MG severity measures, surgical outcomes, and perioperative costs.

Other MG severity outcomes involved the 1‐month postoperative exacerbation rate, MG‐ADL scores at month 3 and 6, QMG scores at month 6, and the changes in prednisone dosage from surgery to month 3.

Surgical outcomes consisted of operative time, intraoperative blood loss, ICU and postoperative hospital stay, assisted respiration duration, pulmonary infection, and POMC, defined as respiratory failure requiring endotracheal intubation or non‐invasive ventilation for over 24 h within 1‐month post‐surgery, or reintubation due to respiratory insufficiency or excessive secretions after extubation [21]. Post‐thymectomy pulmonary infection was identified by clinical manifestation and a new or progressive pulmonary infiltrate on chest imaging and elevated white blood cell counts.

Perioperative costs (3 months pre‐ to post‐surgery) included only direct medical costs, categorized into preoperative fees (only LPE or EFG expenses within 3 months before surgery, excluding oral medications), surgical fees (expenses from thoracic surgery admission to postoperative discharge, excluding LPE and EFG), and postoperative fees (post‐surgical expenses of LPE, EFG, and other biologics such as eculizumab or CD20 monoclonal antibodies). Because of marked price differences before and after reimbursement, LPE and EFG costs were analyzed by both original and post‐reimbursement standards; all other costs were based on original costs only. Original LPE expenses were extracted from transfusion/blood purification center entries in hospitalization bills, and the original price of EFG was set at 5608 CNY per 400 mg. For post‐reimbursement costs, out‐of‐pocket LPE was calculated as original price × patient's co‐payment ratio, while EFG and other biologics were reimbursed in accordance with national medical insurance policies and individual insurance types.

We also explored factors associated with 3‐month postoperative QMG scores and identified risk factors for POMC.

### 2.5 Statistical Analyses

Continuous variables were presented as mean ± standard deviation (SD) or median [interquartile range (IQR)] depending on normality assessed by Shapiro–Wilk test, and categorical variables as frequencies (percentages) for crude/matched samples or weighted percentages for weighted samples.

To control confounding, we applied propensity score‐based overlap weighting to create a balanced pseudo‐population and minimize extreme propensity scores [22, 23]. Variable selection integrated clinical expertise and statistical criteria, including demographics, disease history, preoperative treatments, comorbidities, thymic pathology, and surgical parameters. Covariate balance was quantified using standardized mean differences (SMD), with SMD > 0.2 indicating imbalance. To avoid omitting important confounders in small samples, we used a conservative *p*‐value threshold of < 0.2 for associations with treatment and outcome to classify potential confounders. Clinically important variables were also considered. Multiple candidate models were compared using the Akaike Information Criterion (AIC), and collinearity was assessed via variance inflation factors (VIF > 5 problematic). The final model for Oral IS vs. RACT comparison included 5 key variables: QMG scores at surgery, previous prednisone use, thymic pathology, chronic respiratory disorder, and MG duration; for LPE vs. EFG, it included 3 variables: QMG scores before RACT, preoperative prednisone use, and concomitant autoimmunity.

Missing data were handled by multiple imputation (mice package, 5 datasets, 10 iterations). Average overlap weights across the imputed datasets were calculated, and covariate balance was reassessed using SMD.

Weighted outcome measures were obtained using the survey package: continuous outcomes as weighted means with 95% confidence intervals (CI), and binary outcomes as weighted proportions with Wilson score CIs [24]. Treatment effects were evaluated using weighted regression (logistic for binary and linear for continuous outcomes). Longitudinal QMG and MG‐ADL scores were compared via generalized estimating equations (GEE). Results from 5 imputed datasets were pooled using Rubin's rules [25, 26]. For continuous outcomes and risk differences, we combined point estimates and standard errors across imputations and calculated *p*‐values using the *t*‐distribution with Barnard‐Rubin degrees of freedom, ensuring consistency between confidence intervals and *p*‐values. For odds ratios, pooling was performed on the log scale and similarly back‐transformed, with *p*‐values derived from the t‐distribution.

For exploratory analyses, we used a liberal *p*‐value threshold of < 0.1 in univariable analysis to select candidate variables for backward multivariable regression to avoid missing potentially relevant predictors. For significant continuous variables (*p* < 0.05) in the final model, receiver operating characteristic (ROC) analysis identified optimal cut‐off points.

A two‐sided *p* < 0.05 was considered significant. All analyses and figures were generated using R version 4.4.1.

### 2.6 Sensitivity Analysis

For sensitivity analysis, conventional 1:1 propensity score matching replaced overlap weighting to adjust for baseline differences, using the same variable sets and a caliper width of 0.25. Matched outcomes were analyzed with linear and logistic regression.

Furthermore, to assess the robustness of the identified association between treatment groups and the outcomes to potential unmeasured confounders, *E*‐values were computed from Cohen's *d* effect sizes using the standard formula and compared against *E*‐values for the strongest known baseline‐outcome associations to evaluate relative robustness, using the methods of VanderWeele and Ding [27].

To assess the robustness of our imputation strategy, we compared our primary multiple imputation strategy with a more comprehensive imputation model including additional variables.

## 3 Results

### 3.1 Baseline Characteristics

This study analyzed 78 gMG patients who received immunotherapy before thymectomy, including 41 female (52.6%), with a mean age at MG onset of 47.9 ± 11.8 years, median disease duration of 4.00 [2.00, 8.75] months, and median BMI of 23.40 [21.30, 25.53] kg/m^2^. Within 3 months before surgery, 36 patients received only oral immunosuppressants, while 42 underwent RACT (25 LPE, 17 EFG; Table 1). The interval between last preoperative RACT and thymectomy was 4.0 [2.0, 14.0] days for EFG and 22.0 [15.5, 40.0] days for LPE.

**TABLE 1:** Baseline characteristics of patients treated with RACT vs. Oral IS within 3 months before thymectomy.

Variable	Levels	Unweighted				Weighted			
		Oral IS	RACT	*p*	SMD	Oral IS	RACT	*p*	SMD
*n*		36	42						
Sex, *n* (%) or %	Female Male	22 (61.1%) 14 (38.9%)	19 (45.2%) 23 (54.8%)	0.162	0.322	58.8% 41.2%	43.3% 56.7%	0.240	0.314
BMI (kg/m^2^)		23.53 [22.38, 25.15]	23.21 [20.93, 26.03]	0.548	0.045	24.34 ± 2.38	23.47 ± 3.83	0.258	0.272
Age of onset (y)		46.8 ± 11.4	48.9 ± 12.1	0.435	0.179	48.0 ± 10.1	47.7 ± 12.0	0.915	0.026
MG Duration (months)		4.50 [3.00, 11.00]	4.00 [2.00, 8.00]	0.389	0.217	8.28 ± 14.16	8.28 ± 15.36	1.000	< 0.001
Previous Crisis, *n* (%) or %		1 (2.8%)	1 (2.4%)	1.000	0.025	4.1%	3.3%	0.874	0.043
AChR‐ab positive, *n* (%) or %		29 (96.7%)	41 (100.0%)	0.423	0.263	94.3%	100.0%	0.261	0.347
Titin‐ab positive, *n* (%) or %		8 (27.6%)	7 (17.5%)	0.316	0.243	25.0%	17.5%	0.493	0.186
RyR‐ab positive, *n* (%) or %		4 (13.8%)	7 (17.9%)	0.747	0.114	19.7%	14.0%	0.586	0.151
Previous Pred, *n* (%) or %		30 (83.3%)	24 (57.1%)	0.012	0.598	72.5%	72.5%	1.000	< 0.001
Previous TAC, *n* (%) or %		18 (50.0%)	22 (52.4%)	0.834	0.048	49.0%	48.4%	0.966	0.011
Previous MMF, *n* (%) or %		2 (5.6%)	4 (9.5%)	0.681	0.151	1.3%	9.9%	0.011	0.382
Previous AZA, *n* (%) or %		2 (5.6%)	0 (0.0%)	0.210	0.343	1.9%	0.0%	0.243	0.195
TAC at surgery, *n* (%) or %		17 (47.2%)	19 (45.2%)	0.861	0.040	49.0%	39.7%	0.474	0.186
MMF at surgery, *n* (%) or %		1 (2.8%)	4 (9.5%)	0.366	0.284	0.9%	9.9%	0.009	0.407
Pred dose at surgery (mg/d)		20.00 [3.75, 20.00]	20.00 [0.00, 23.75]	0.695	0.138	13.57 ± 10.58	15.78 ± 11.34	0.421	0.201
Hypertension, *n* (%) or %		8 (22.2%)	7 (16.7%)	0.535	0.141	26.4%	13.1%	0.197	0.339
Diabetes, *n* (%) or %		6 (16.7%)	4 (9.5%)	0.500	0.213	24.0%	8.9%	0.117	0.418
Autoimmunity, *n* (%) or %		4 (11.1%)	4 (9.5%)	1.000	0.052	5.6%	11.5%	0.327	0.213
Chronic respiratory disorders, *n* (%) or %		5 (13.9%)	13 (31.0%)	0.075	0.418	19.9%	19.9%	1.000	< 0.001
Pathology, *n* (%) or %	Cyst Hyperplasia Thymoma Undefined	0 (0.0%) 7 (19.4%) 28 (77.8%) 1 (2.8%)	1 (2.4%) 1 (2.4%) 40 (95.2%) 0 (0.0%)	0.012	0.671	0.0% 5.9% 94.1% 0.0%	0.0% 5.9% 94.1% 0.0%	1.000	< 0.001
Thymoma WHO type, *n* (%) or %	A + AB+B1 B2 + B3 Others Non‐thymoma	6 (16.7%) 21 (58.3%) 1 (2.8%) 8 (22.2%)	8 (19.0%) 31 (73.8%) 1 (2.4%) 2 (4.8%)	0.097	0.534	21.6% 68.4% 4.1% 5.9%	21.8% 68.7% 3.6% 5.9%	1.000	0.022
Surgery, *n* (%) or %	OPEN RATS VATS	2 (5.6%) 8 (22.2%) 26 (72.2%)	4 (9.5%) 5 (11.9%) 33 (78.6%)	0.421	0.301	5.5% 16.4% 78.1%	13.2% 12.6% 74.2%	0.593	0.279
Radiation, *n* (%) or %		18 (50.0%)	16 (38.1%)	0.291	0.242	56.1%	37.1%	0.143	0.389
QMG scores before thymectomy		7.92 ± 2.79	9.12 ± 3.09	0.078	0.408	8.58 ± 2.40	8.58 ± 2.91	1.000	< 0.001
MG‐ADL scores before thymectomy		1.50 [0.00, 4.00]	2.00 [1.00, 3.00]	0.639	0.038	2.61 ± 1.97	2.04 ± 1.52	0.204	0.324

*Note:* Effective sample sizes: 32.9 in Oral IS group; 27.6 in RACT group.

Abbreviations: Ab, antibody; AChR, acetylcholine receptor; AZA, Azathioprine; BMI, body mass index; IS, immunosuppressants; MG‐ADL, Activities of Daily Living of myasthenia gravis; MMF, Mycophenolate mofetil; OPEN, open thoracotomy; Pred, prednisone; QMG, Quantitative Myasthenia Gravis; RACT, rapid antibody clearance therapy; RATS, robot‐assisted thoracoscopic surgery; RyR, Ryanodine Receptor; SMD, standardized mean differences; TAC, tacrolimus; VATS, video‐assisted thoracoscopic surgery.

### 3.2 Covariate Balance After Overlap Weighting

**FIGURE 2:**
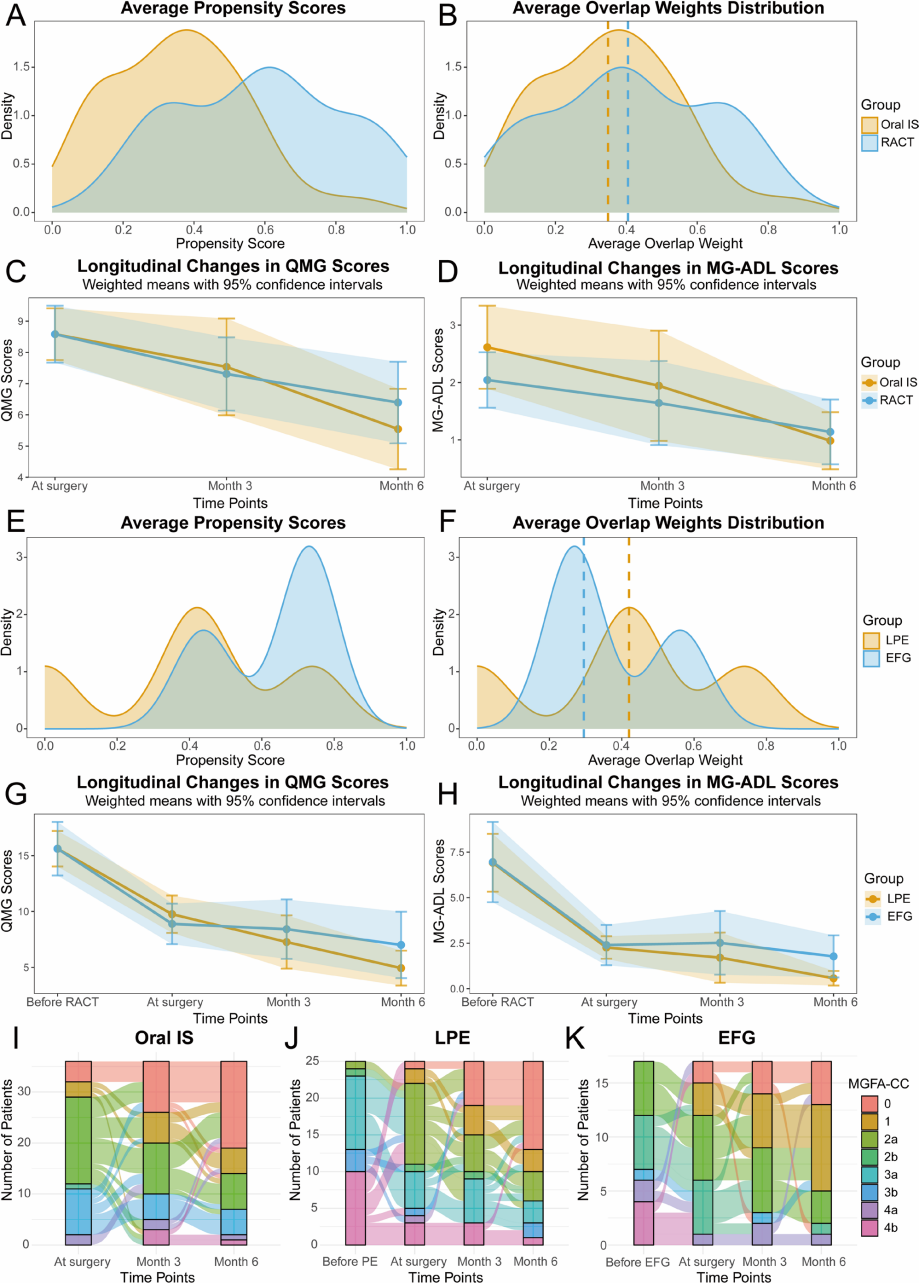
Preoperative immunotherapy strategies and their impact on post‐thymectomy outcomes in gMG patients. (A–D) Comparison between oral immunosuppressants (Oral IS) and rapid antibody clearance therapy (RACT) groups. (A) Density plots of the propensity scores before overlap weighting for Oral IS vs. RACT group. (B) Overlap weight distribution of Oral IS vs. RACT groups. (C, D) Overlap‐weighted mean of QMG and MG‐ADL score trajectories for the Oral IS and RACT groups from surgery to 6 months postoperatively, analyzed using generalized estimating equations (GEE). (E–H) Comparison between lymphoplasmapheresis (LPE) and Efgartigimod (EFG) groups. (E) Density plots of the propensity scores for the LPE vs. EFG comparison before overlap weighting. (F) The distribution of overlap weights for the LPE and EFG groups. (G,H) Overlap‐weighted mean QMG and MG‐ADL score trajectories for the LPE and EFG groups over time, analyzed using GEE. (I–K) MGFA‐CC changes for Oral IS, LPE, and EFG groups.

Propensity score distributions showed sufficient overlap between the Oral IS and RACT groups, supporting the use of overlap weighting (Figure 2A). After weighting based on QMG scores at surgery, prior prednisone use, thymic pathology, chronic respiratory disorders, and MG duration, baseline covariates were well balanced (Table 1, Figure S1A). The median weights were 0.348 (Oral IS) and 0.405 (RACT), yielding effective sample sizes of 32.9 and 27.6, respectively (Figure 2B). Although QMG scores were nearly identical after weighting, the RACT group had a higher proportion of mycophenolate mofetil (MMF) use (Table 1).

For the LPE versus EFG comparison, only patients undergoing VATS were included (16 LPE and 17 EFG). Overlap weighting based on previous prednisone use, QMG scores before RACT, and autoimmunity achieved covariates balance (Figure S2A, Table 2). Median weights were 0.420 (LPE) and 0.295 (EFG), with effective sample sizes of 11.1 and 14.8, respectively (Figure 2F). After weighting, mean pre‐RACT QMG scores were identical (15.62 in both groups) and decreased markedly thereafter. At surgery, the LPE group exhibited slightly higher QMG scores than the EFG group, and a higher proportion of EFG patients had received MMF.

**TABLE 2:** Baseline characteristics of patients treated with EFG vs. LPE within 3 months before thymectomy.

Variable	Levels	Unweighted				Weighted			
		LPE	EFG	*p*	SMD	LPE	EFG	*p*	SMD
*n*		16	17						
Sex, *n* (%) or %	Female	9 (56.2%)	8 (47.1%)	0.598	0.185	49.3%	44.3%	0.804	0.101
	Male	7 (43.8%)	9 (52.9%)			50.7%	55.7%		
BMI (kg/m^2^)		23.76 [20.98, 26.80]	23.18 [21.11, 24.14]	0.540	0.317	24.39 ± 4.18	23.15 ± 3.24	0.368	0.330
Age of onset (y)		45.8 ± 10.2	51.1 ± 13.7	0.218	0.440	42.9 ± 9.7	50.4 ± 13.1	0.091	0.654
MG duration (months)		3.50 [2.00, 6.50]	4.00 [2.00, 7.00]	0.956	0.260	7.96 ± 10.91	13.30 ± 26.01	0.536	0.268
Previous crisis, *n* (%) or %		1 (6.2%)	0 (0.0%)	0.485	0.365	0.0%	0.0%	0.379	< 0.001
AChR‐ab, *n* (%) or %		15 (100.0%)	17 (100.0%)	NA	< 0.001	100.0%	100.0%	NA	< 0.001
Titin‐ab, *n* (%) or %		1 (6.7%)	5 (29.4%)	0.178	0.619	6.6%	28.5%	0.133	0.602
RyR‐ab, *n* (%) or %		1 (7.1%)	4 (23.5%)	0.344	0.467	6.6%	21.2%	0.261	0.432
Previous Pred, *n* (%) or %		11 (68.8%)	6 (35.3%)	0.055	0.711	53.2%	53.2%	1.000	< 0.001
Previous TAC, *n* (%) or %		9 (56.2%)	9 (52.9%)	0.849	0.066	72.4%	57.8%	0.432	0.310
Previous MMF, *n* (%) or %		1 (6.2%)	2 (11.8%)	1.000	0.194	0.0%	8.4%	< 0.001	0.427
Previous AZA, *n* (%) or %		0 (0.0%)	0 (0.0%)	NA	< 0.001	0%	0%	NA	< 0.001
TAC at surgery, *n* (%) or %		7 (43.8%)	8 (47.1%)	0.849	0.066	59.5%	48.2%	0.571	0.227
MMF at surgery, *n* (%) or %		1 (6.2%)	2 (11.8%)	1.000	0.194	0.0%	8.4%	< 0.001	0.427
Pred dose at surgery (mg/d)		20.00 [0.00, 25.00]	0.00 [0.00, 20.00]	0.095	0.555	13.33 ± 13.31	13.38 ± 11.78	0.991	0.005
Hypertension, *n* (%) or %		2 (12.5%)	2 (11.8%)	1.000	0.023	12.0%	13.0%	0.946	0.031
Diabetes, *n* (%) or %		0 (0.0%)	1 (5.9%)	1.000	0.354	0.0%	4.4%	0.337	0.302
Autoimmunity, *n* (%) or %		4 (25.0%)	0 (0.0%)	0.044	0.816	0.0%	0.0%	0.224	< 0.001
Chronic respiratory disorder, *n* (%) or %		6 (37.5%)	5 (29.4%)	0.622	0.172	38.7%	33.2%	0.778	0.115
Pathology, *n* (%) or %	Non‐thymoma Thymoma	1 (6.2%) 15 (93.8%)	1 (5.9%) 16 (94.1%)	1.000	0.015	6.7% 93.3%	4.9% 95.1%	0.821	0.079
Thymoma WHO type, *n* (%) or %	A + AB+B1 B2 + B3 Others	5 (31.2%) 9 (56.2%) 2 (12.5%)	2 (11.8%) 14 (82.4%) 1 (5.9%)	0.328	0.592	41.5% 51.8% 6.7%	12.9% 82.2% 4.9%	0.223	0.707
Radiation, *n* (%) or %		7 (43.8%)	3 (17.6%)	0.141	0.590	39.4%	21.9%	0.359	0.386
QMG scores before RACT		16.69 ± 3.34	15.71 ± 5.05	0.518	0.229	15.62 ± 2.95	15.62 ± 4.97	1.000	< 0.001
MG‐ADL scores before RACT		7.44 ± 2.56	7.24 ± 4.82	0.881	0.052	6.91 ± 2.81	6.95 ± 4.65	0.978	0.010
QMG scores before surgery		9.00 [7.75, 10.00]	8.00 [6.00, 11.00]	0.276	0.325	9.76 ± 2.93	8.89 ± 3.50	0.501	0.269
MG‐ADL scores before surgery		2.50 [2.00, 3.00]	2.00 [1.00, 4.00]	0.397	0.124	2.26 ± 1.15	2.40 ± 2.12	0.838	0.079

*Note:* Effective sample sizes: 11.1 in LPE group; 14.8 in EFG group.

Abbreviations: Ab, antibody; AChR, acetylcholine receptor; AZA, Azathioprine; BMI, body mass index; EFG, Efgartigimod; LPE, lymphoplasmapheresis; MG‐ADL, Activities of Daily Living of myasthenia gravis; MMF, Mycophenolate mofetil; OPEN, open thoracotomy; Pred, prednisone; QMG, Quantitative Myasthenia Gravis; RATS, robot‐assisted thoracoscopic surgery; RyR, Ryanodine Receptor; SMD, standardized mean differences; TAC, tacrolimus; VATS, video‐assisted thoracoscopic surgery.

### 3.3 Primary Outcome

No significant difference was observed in 3‐month post‐thymectomy QMG scores between any compared groups (Table 3). For RACT vs. Oral IS, overlap‐weighted mean QMG scores were 7.31 (95% CI: 5.79 to 8.83) and 7.54 (95% CI: 5.79 to 9.28), respectively, with a non‐significant between‐group difference of −0.23 (95% CI: −2.99 to 2.54; *p* = 0.831). Similarly, for EFG vs. LPE, scores were 8.42 (95% CI: 6.12 to 10.71) for EFG and 7.27 (95% CI: 5.10 to 9.44) for LPE (difference 1.15, 95% CI: −3.97 to 6.26; *p* = 0.567).

**TABLE 3:** Primary outcomes.

Primary outcomes	(1) Mean (95% CI)	(0) Mean (95% CI)		B (95% CI) (1–0)	*p*
RACT (1) vs. Oral IS (0)					
QMG scores at Month 3 after thymectomy	7.31 (5.79, 8.83)	7.54 (5.79, 9.28)	Primary comparison	−0.23 (−2.99, 2.54)	0.831
			Sensitivity analysis	−0.13 (−2.63, 2.37)	0.917
EFG (1) vs. LPE (0)					
QMG scores at Month 3 after thymectomy	8.42 (6.12, 10.71)	7.27 (5.10, 9.44)	Primary comparison	1.15 (−3.97, 6.26)	0.567
			Sensitivity analysis	0.30 (−4.09, 4.69)	0.887

Abbreviations: EFG, Efgartigimod; IS, immunosuppressants; LPE, lymphoplasmapheresis; QMG, Quantitative Myasthenia Gravis; RACT, rapid antibody clearance therapy.

### 3.4 Oral IS vs. RACT

No significant differences emerged in MG severity outcomes, surgical outcomes, and financial outcome between the RACT and Oral IS groups (Table S1), including 1‐month clinical worsening rate (28.1% vs. 23.5%, *p* = 0.704), 6‐month QMG scores (6.39 vs. 5.54, *p* = 0.417), operative time (124.26 vs. 105.45 min, *p* = 0.266), blood loss (88.52 vs. 57.46 mL, *p* = 0.366), and hospital stay (5.70 vs. 6.77 days, *p* = 0.427).

Longitudinal GEE analysis revealed a significant time effect on QMG scores (Figure 2C, Table S2), with scores decreasing by −1.05 points (95% CI: −2.81 to 0.71; *p* = 0.243) at 3 months and −3.04 points (95% CI: −4.57 to −1.51; *p* < 0.001) at 6 months relative to baseline. No significant treatment effect or group‐by‐time interaction was observed. MG‐ADL scores showed a comparable longitudinal pattern (Figure 2D, Table S2).

### 3.5 LPE vs. EFG

The 1‐month clinical worsening rate was 43.3% in the EFG group and 26.1% in the LPE group, although this difference did not reach statistical significance (*p* = 0.412). At 6 months, there were no significant between‐group differences in QMG scores (EFG: 7.01 vs. LPE: 4.93, *p* = 0.296) and MG‐ADL scores (EFG: 1.77 vs. LPE: 0.57, *p* = 0.604). Longitudinal analysis confirmed significant improvement in QMG scores over time in both groups (*p* < 0.001), with no significant group‐by‐time interaction (Figure 2G,H; Table S2).

In the EFG versus LPE comparison, no significant differences were observed for surgical outcomes including POMC (9.6% vs. 7.3%, *p* = 0.853), operative time (88.72 min vs. 109.09 min, *p* = 0.143), and blood loss (20.61 mL vs. 28.47 mL, *p* = 0.238; Table 4).

**TABLE 4:** Secondary outcomes among patients who received EFG vs. LPE within 3 months before thymectomy (LPE as reference).

Outcomes	EFG weighted	LPE weighted		B (95% CI)	OR (95% CI)	*p*
*MG severity outcomes*						
Exacerbated within 1 month after surgery	43.3% (22.2%, 67.2%)	26.1% (9.1%, 55.3%)	Primary comparison	—	2.17 (0.19, 25.19)	0.412
			Sensitivity analysis	—	2.33 (0.38, 16.29)	0.365
MG‐ADL scores at Month 3 after thymectomy	2.52 (0.66, 4.38)	1.71 (0.06, 3.36)	Primary comparison	0.81 (−2.37, 3.99)		0.518
			Sensitivity analysis	0.90 (−1.65, 3.45)		0.468
QMG scores at Month 6 after thymectomy	7.01 (4.58, 9.44)	4.93 (3.17, 6.69)	Primary comparison	2.08 (−2.73, 6.88)		0.296
			Sensitivity analysis	1.40 (−2.99, 5.79)		0.511
MG‐ADL scores at Month 6 after thymectomy	1.77 (0.26, 3.29)	0.57 (−0.32, 1.46)	Primary comparison	1.10 (−3.28, 5.48)		0.604
			Sensitivity analysis	1.30 (−0.33, 2.93)		0.111
Pred dose change (mg/d)	0.82 (−1.98, 3.62)	1.48 (−2.35, 5.30)	Primary comparison	−0.66 (−12.67, 11.36)		0.887
			Sensitivity analysis	4.75 (−5.62, 15.12)		0.348
*Surgery outcomes*						
POMC	9.6% (2.2%, 33.6%)	7.3% (1.1%, 35.4%)	Primary comparison	—	1.35 (0.02, 85.78)	0.853
			Sensitivity analysis	—	1.00 (0.04, 28.00)	1.000
Operative time (min)	88.72 (84.37, 93.06)	109.09 (102.87, 115.31)	Primary comparison	−20.37 (−51.50, 10.75)		0.143
			Sensitivity analysis	−22.60 (−50.15, 4.95)		0.102
Blood loss (mL)	20.61 (16.85, 24.37)	28.47 (24.39, 32.54)	Primary comparison	−7.85 (−23.59, 7.88)		0.238
			Sensitivity analysis	−13.00 (−26.01, 0.01)		0.050
ICU length of stay (hours)	7.50 (2.60, 12.41)	11.07 (7.59, 14.56)	Primary comparison	−3.57 (−23.06, 15.92)		0.638
			Sensitivity analysis	−3.16 (−19.38, 13.06)		0.687
Assisted breathing time (hours)	6.47 (1.96, 10.98)	4.24 (1.60, 6.87)	Primary comparison	2.23 (−13.30, 17.76)		0.711
			Sensitivity analysis	1.24 (−12.00, 14.48)		0.846
Pulmonary infection	9.6% (2.2%, 33.6%)	26.1% (9.1%, 55.3%)	Primary comparison	—	0.30 (0.01, 10.03)	0.369
			Sensitivity analysis	—	0.26 (0.01, 2.54)	0.284
Postoperative hospitalization days	4.45 (2.32, 6.59)	6.05 (4.23, 7.87)	Primary comparison	−1.60 (−5.66, 2.46)		0.336
			Sensitivity analysis	−1.40 (−4.78, 1.98)		0.395
*Financial outcomes*						
Surgery cost (CNY)	26813.42 (26733.15, 26893.69)	32168.33 (32084.62, 32252.04)	Primary comparison	−5354.91 (−12235.02, 1525.20)		0.097
			Sensitivity analysis	−4985.05 (−10960.20, 990.10)		0.097
Preoperative cost (original) (CNY)	24554.13 (24428.48, 24679.78)	10053.72 (9996.11, 10111.33)	Primary comparison	14500.41 (2840.28, 26160.54)		0.026
			Sensitivity analysis	13814.35 (874.82, 26753.89)		0.038
Preoperative cost (after reimbursement) (CNY)	9505.67 (9427.21, 9584.12)	6666.07 (6617.82, 6714.32)	Primary comparison	2839.60 (−1916.47, 7595.67)		0.173
			Sensitivity analysis	2773.71 (−2526.80, 8074.22)		0.286
Postoperative cost (original) (CNY)	21137.40 (20967.89, 21306.92)	5118.25 (5009.56, 5226.94)	Primary comparison	16019.15 (−6436.22, 38474.53)		0.119
			Sensitivity analysis	20533.19 (188.33, 40878.05)		0.048
Postoperative cost (after reimbursement) (CNY)	9348.68 (9229.80, 9467.56)	3489.29 (3391.47, 3587.10)	Primary comparison	5859.40 (−6474.94, 18193.74)		0.258
			Sensitivity analysis	7647.97 (−4188.06, 19484.01)		0.191
Perioperative costs (original) (CNY)	73323.39 (73112.41, 73534.36)	47340.30 (47237.28, 47443.31)	Primary comparison	25983.09 (−7087.04, 59053.22)		0.095
			Sensitivity analysis	31605.69 (2277.63, 60933.75)		0.036
Perioperative costs (after reimbursement) (CNY)	45995.14 (45841.79, 46148.49)	42323.68 (42229.80, 42417.56)	Primary comparison	3671.46 (−14478.68, 21821.59)		0.578
			Sensitivity analysis	6333.91 (−9442.57, 22110.39)		0.410

*Note:* Costs are reported in Chinese Yuan. Surgical costs are presented as un‐reimbursed (original) values, as the reimbursement rate for surgical expenses was identical across all groups, resulting in no between‐group differences in net surgical costs after reimbursement. Primary comparison: balancing LPE and EFG groups with overlap weighting; sensitivity analysis: balancing LPE and EFG groups with propensity score matching.

Abbreviations: EFG, Efgartigimod; ICU, intensive care unit; LPE, lymphoplasmapheresis; MG‐ADL, Activities of Daily Living of myasthenia gravis; POMC, postoperative myasthenic crisis; Pred, prednisone; QMG, Quantitative Myasthenia Gravis.

Regarding costs, the EFG approach was associated with significantly higher original cost of preoperative preparation (mean difference: 14500.41 CNY, 95% CI: 2840.28 to 26160.54; *p* = 0.026). In the entire cohort, the average costs of per LPE session and 400 mg EFG were 5850.62 CNY and 5608 CNY, respectively, dropping to 4044.23 CNY and 2172.55 CNY after reimbursement. However, all patients with EFG treatment in our cohort were covered by medical insurance reimbursement, after which the preoperative (*p* = 0.173) and perioperative costs (*p* = 0.578) were similar between groups (Table 4).

### 3.6 Exploration Outcomes

In univariate analysis of factors associated with 3‐month postoperative QMG scores, only the QMG scores at surgery showed a significant association (*p* < 0.001; Table S3).

For POMC, univariate analysis identified higher BMI (*p* = 0.003), chronic respiratory disease (*p* = 0.023), and higher preoperative QMG scores (*p* = 0.014) as significant risk factors, while female sex and tacrolimus use (both prior and at surgery) reduced risk (*p* = 0.071, 0.082, and 0.053, respectively). Multivariable backward stepwise regression confirmed BMI (OR = 1.38, 95% CI: 1.12 to 1.83, *p* = 0.007) and preoperative QMG scores (OR = 1.59, 95% CI: 1.15 to 2.50, *p* = 0.013) as independent predictors (Table S4).

A combined model (BMI, preoperative QMG scores, tacrolimus use, gender) showed excellent POMC discrimination (AUC = 0.937), with 77.8% sensitivity and 98.6% specificity at the optimal cutoff. Individually, BMI (AUC = 0.726, cutoff 27.99 kg/m^2^) and preoperative QMG scores (AUC = 0.742, cutoff 8.5) showed moderate predictive ability (Figure S3).

### 3.7 Sensitivity Analysis

Propensity score‐matched analysis (23 RACT vs. Oral IS pairs; 10 EFG vs. LPE pairs) confirmed no significant difference in 3‐month QMG scores across groups, consistent with primary analyses (Tables 3, S5, S6).

However, in the LPE vs. EFG comparison, matched analysis revealed differences between the two groups for preoperative expenses before reimbursement (*p* = 0.038), postoperative expenses before reimbursement (*p* = 0.048), and perioperative expenses before reimbursement (*p* = 0.036). No significant differences were observed for blood loss (*p* = 0.050), operative time (*p* = 0.102), or surgical cost (*p* = 0.097; Table 4).

For significant and marginally significant treatment effects, *E*‐value sensitivity analyses revealed that at the point estimate level, surgery costs and perioperative costs (before reimbursement) showed treatment effect *E*‐values exceeding those of the strongest known baseline variable associations (surgery cost: 2.19 vs. 2.04; perioperative costs (before reimbursement): 2.12 vs. 1.90). In contrast, conservative estimates based on confidence interval boundaries showed lower treatment effect *E*‐values relative to baseline association *E*‐values (Table S7).

Additionally, results remained consistent across multiple imputation approaches, supporting the robustness of the primary analysis (Figures S1B,C and S2B,C).

## 4 Discussion

Preoperative administration of EFG or LPE effectively reduces QMG scores, with no significant postoperative medium‐term effectiveness and safety differences between RACT and Oral IS alone. As the first real‐world study to evaluate EFG and LPE as preoperative therapies, we found no statistically significant difference between the two regimens in improving acute MG symptoms. While this finding does not establish equivalence, it suggests that EFG warrants further investigation as a potential preoperative option.

In severe MG patients, thymectomy following RACT did not increase surgical risks or postoperative exacerbation. RACT rapidly reduced QMG scores preoperatively with shorter preparation time, yielding postoperative improvement similar to Oral IS alone. Although the Oral IS group showed a slightly faster QMG scores reduction, this non‐significant difference may be attributed to their lower proportion of bulbar‐involved patients and more stable immune status. RACT is not routinely recommended for mild MG patients [28]. Given the delayed onset of action of conventional immunosuppressive therapies in MG, early diagnosis and timely thymectomy are critical. Accumulating evidence indicates that preoperative disease duration is a key determinant of thymectomy effectiveness, with superior surgical outcomes when thymectomy is performed within 12 months of symptom onset or within 3 years in late‐onset MG [29, 30, 31]. These findings suggest that prompt and effective treatment in the early stages of the disease may prevent irreversible damage to the neuromuscular junction and impede the migration of long‐lived plasma cells from the thymus to the periphery, ultimately leading to more favorable long‐term results [31].

A pioneering “2 + 2 regimen” of EFG for TAMG demonstrated rapid preoperative optimization and stable postoperative recovery, forming the foundation for this real‐world investigation [17]. However, in clinical practice, the decision on the number of EFG infusions and LPE procedures was guided by the patient's MG severity and treatment response. For this study, we quantified these individualized decisions by analyzing preoperative costs. The longer preoperative interval in the LPE group (median 22 days) versus the EFG (4 days) reflects distinct onset kinetics. In the ADAPT trial, EFG demonstrated clinically meaningful improvement as early as 1 week after the first infusion, with the effect lasting approximately 8 weeks per treatment cycle [32]. In contrast, LPE typically requires 1–14 days to achieve therapeutic effect, which can be maintained for 3–4 weeks [33, 34, 35]. Both intervals are well within the 3‐month preoperative window and reflect real‐world clinical practice.

Our real‐world data indicate that a symptom‐guided perioperative course of EFG showed no statistically significant difference in clinical effectiveness and overall cost when compared to LPE. The higher pre‐reimbursement costs of EFG were offset by China's medical insurance policy, achieving cost equivalence; thus, regimen selection in global practice should be guided by local reimbursement frameworks. Although preoperative effectiveness was comparable, the EFG group had a higher short‐term postoperative exacerbation rate than the LPE group, whereas LPE had lower QMG and MG‐ADL scores at 6 months. However, none of these differences reached statistical significance. Given the exploratory nature of this study (no pre‐specified sample size calculation), these findings should be interpreted with caution. Nevertheless, they suggest that closer postoperative monitoring may be considered for patients receiving preoperative EFG.

These postoperative observations may be primarily attributed to mechanistic differences between LPE and EFG. Specifically, while the full mechanism of LPE remains incompletely understood, it achieves broad and rapid clearance of circulating pathogenic factors, including AChR antibodies, inflammatory cytokines (e.g., IL‐17, IFN‐γ, IL‐12p70), and immune cell subsets, without inducing the antibody rebound associated with conventional PE [13, 14, 18, 36]. In contrast, although EFG blocks FcRn‐mediated IgG recycling [15], it has been linked to increased total B cells and antibody‐secreting cells, along with non‐pathogenic antibody production. It did not significantly reduce pathogenic T‐bet+ B cells, and a subset of T cells remained aberrantly activated [37, 38]. Second, study limitations may also have influenced these findings, including a limited sample size and suboptimal EFG response in certain TAMG patients. Some cases with thymoma recurrence showed poor response to EFG and experienced significant postoperative worsening, aligning with previous reports [39, 40, 41]. Mechanistically, the persistence and peripheral escape of aberrant Th cells post‐resection (driving AChR‐Ab production by B cells) or AChR‐Ab overshoot may explain EFG's suboptimal effectiveness in these TAMG patients [39, 42, 43], highlighting the need for further studies to evaluate EFG's role in TAMG.

In this study, the EFG group had shorter operative time and reduced blood loss compared to the LPE group, although these differences were not statistically significant. The LPE group incurred higher thymectomy‐related costs and longer hospital stays, likely due to more frequent postoperative ICU transfers—a practice reflective of earlier critical care standards. With advances in surgical technique and multidisciplinary care, routine ICU monitoring has decreased [44], and this cost disparity is expected to diminish accordingly. EFG provides disease control comparable to LPE at similar post‐reimbursement cost, while eliminating plasma supply requirement and offering greater clinical flexibility [45, 46]. Furthermore, recent evidence indicates that EFG outperforms IVIG in symptom improvement, crisis prevention, and safety, supporting its role as a preferred therapeutic strategy [40].

To further explore factors associated with postoperative prognosis, we found that preoperative QMG scores were significantly associated with both the 3‐month QMG scores and POMC risk. We then constructed a multivariable prediction model incorporating preoperative QMG scores, BMI, gender, and tacrolimus use, which demonstrated robust predictive capacity for post‐thymectomy POMC risk, showing high discriminative accuracy (AUC: 0.937) with exceptional specificity that minimizes false positives (98.6%) and adequate sensitivity (77.8%) for clinical utility. These findings align with existing literature: Leuzzi et al. identified high Osserman stages (IIB or III–IV), BMI > 28 kg/m^2^, history of myasthenic crisis, symptom duration > 2 years, and prior lung resection as risk factors for POMC [47]. In our study, neither LPE nor EFG affected POMC, consistent with reports on IVIG and PE [47, 48]. To prevent POMC in patients with severe MG, it is still clinically necessary to implement preoperative RACT to reduce the preoperative QMG scores to ≤ 8.

Our study has several limitations. First, despite achieving precise balance in key covariates through overlap weighting, the retrospective single‐center design remains susceptible to selection bias and residual confounding due to observed imbalances in other baseline variables [49]. Second, this is an exploratory study with no pre‐specified non‐inferiority margin. The limited sample size also weakened statistical power and generalizability. The observed non‐significant differences cannot confirm that the two regimens have equivalent efficacy. Third, individualized dosing and frequency of LPE and EFG introduce unaccounted treatment heterogeneity, though this reflects real‐world practice. Finally, the relatively short follow‐up duration (up to 6 months) precludes long‐term conclusions. Thus, our findings are hypothesis‐generating and warrant validation in larger, multi‐center, prospective, controlled studies with longer follow‐up.

## 5 Conclusion

In this exploratory study, preoperative administration of RACT effectively reduced QMG scores, with no significant postoperative medium‐term effectiveness and safety differences between RACT and Oral IS. EFG and LPE showed no statistically significant differences in postoperative medium‐term effectiveness, safety, or post‐reimbursement economic burden for gMG patients undergoing thymectomy. Although pre‐reimbursement costs were higher with EFG, no significant intergroup cost difference was detected after reimbursement, a change attributable to China's medical insurance policy. However, owing to its retrospective nature, limited sample size, and lack of non‐inferiority design, these findings remain preliminary. Larger prospective trials are required for further validation.

## Author Contributions

Q.Z., T.H. and H.Y. were responsible for the study design; Q.Z. performed the data analysis; Q.Z. and T.H. contributed to data validation, drafting, and critical revision of the manuscript. Y.C., K.C. and Y.O. performed patient screening and data collection. Y.O., Z.W., and G.S. helped revise the manuscript. X.D. performed muscle strength test and helped collect clinical data. All authors read and approved the final manuscript.

## Funding

This work was supported by the National Natural Science Foundation of China (grant numbers 82171399, 82371413, and 82571597).

## Ethics Statement

We confirm that we have read and understood CNS Neuroscience & Therapeutics' position on issues involved in ethical publication and affirm that this report is consistent with those guidelines. This study was approved by the Ethics Board of Xiangya Hospital, Central South University (approval number: 2025112118).

## Consent

Informed consent for publication was waived by the ethics committee since this was a retrospective study. All patient identifiers were removed to protect confidentiality and anonymity.

## Conflicts of Interest

The authors declare no conflicts of interest.

## Supporting information


**Table S1:** Secondary outcomes among patients who received RACT vs. Oral IS within 3 months before thymectomy (Oral IS as reference). This table summarizes the MG severity, surgical, and financial outcomes for RACT and Oral IS group, with overlap weighting as the primary comparison and propensity score matching as the sensitivity analysis.
**Table S2:** Generalized estimating equations (GEE) analysis of QMG and MG‐ADL scores for models of RACT vs. Oral IS and models of EFG vs. LPE.
**Table S3:** Factors associated with QMG scores at month 3 after thymectomy. Univariable linear regression analyses were performed to identify factors associated with 3‐month postoperative QMG scores.
**Table S4:** Factors associated with POMC. Univariable and multivariable logistical regression analyses were performed to identify factors independently associated with POMC.
**Table S5:** Baseline characteristics of patients with RACT vs. Oral IS within 3 months before thymectomy before and after propensity score matching.
**Table S6:** Baseline characteristics of patients with EFG vs. LPE within 3 months before thymectomy before and after propensity score matching.
**Table S7:** E‐value analyses for EFG vs. LPE outcomes. E‐values were calculated for treatment effects that were marginally significant. The table shows point‐estimate and conservative E‐values, along with the strongest baseline‐outcome E‐value for context. Larger E‐values imply greater robustness to unmeasured confounding.
**Figure S1:** Covariate balancing, missing data patterns, and sensitivity analysis for the Oral IS vs. RACT comparison.
**Figure S2:** Covariate balancing, missing data patterns, and sensitivity analysis for the LPE vs. EFG comparison.
**Figure S3:** ROC curves for POMC prediction.

## Data Availability

The data that support the findings of this study are available on request from the corresponding author. The data are not publicly available due to privacy or ethical restrictions.
