# Supplementary material for: Efgartigimod Versus Lymphoplasmapheresis as Preoperative Rapid Antibody‐Clearing Therapies for Thymectomy in Generalized Myasthenia Gravis: Effectiveness, Safety and Cost Outcomes Compared to Conventional Preparation
Source: CNS Neurosci Ther. 2026 Jun 19;32(6):e70993. doi: 10.1002/cns.70993 (PMC13282466; doi:10.1002/cns.70993)
Supplement: Supplementary file 1 — Table S1: Secondary outcomes among patients who received RACT vs. Oral IS within 3 months before thymectomy (Oral IS as reference). This table summarizes the MG severity, surgical, and financial outcomes for RACT and Oral IS group, with overlap weighting as the primary comparison and propensity score matching as the sensitivity analysis. Table S2: Generalized estimating equations (GEE) analysis of QMG and MG‐ADL scores for models of RACT vs. Oral IS and models of EFG vs. LPE. Table S3: Factors associated with QMG scores at month 3 after thymectomy. Univariable linear regression analyses were performed to identify factors associated with 3‐month postoperative QMG scores. Table S4: Factors associated with POMC. Univariable and multivariable logistical regression analyses were performed to identify factors independently associated with POMC. Table S5: Baseline characteristics of patients with RACT vs. Oral IS within 3 months before thymectomy before and after propensity score matching. Table S6: Baseline characteristics of patients with EFG vs. LPE within 3 months before thymectomy before and after propensity score matching. Table S7: E‐value analyses for EFG vs. LPE outcomes. E‐values were calculated for treatment effects that were marginally significant. The table shows point‐estimate and conservative E‐values, along with the strongest baseline‐outcome E‐value for context. Larger E‐values imply greater robustness to unmeasured confounding. Figure S1: Covariate balancing, missing data patterns, and sensitivity analysis for the Oral IS vs. RACT comparison. Figure S2: Covariate balancing, missing data patterns, and sensitivity analysis for the LPE vs. EFG comparison. Figure S3: ROC curves for POMC prediction. [file CNS-32-e70993-s001.docx]

**Supplementary materials**

**Efgartigimod versus Lymphoplasmapheresis as Preoperative Rapid Antibody-Clearing Therapies for** **Thymectomy in Generalized Myasthenia Gravis: Effectiveness, Safety and Cost Outcomes Compared to Conventional Preparation**

Table S1. Secondary outcomes among patients who received RACT vs. Oral IS within 3 months before thymectomy (Oral IS as reference).

| Outcomes | RACT  Weighted | Oral IS  Weighted |  | B (95%CI) | OR (95%CI) | p |
| --- | --- | --- | --- | --- | --- | --- |
| MG severity outcomes |  |  |  |  |  |  |
| Exacerbated within 1 month after surgery | 28.1% (15.6%, 45.2%) | 23.5% (11.5%, 41.9%) | Primary comparison | - | 1.27 (0.24, 6.73) | 0.704 |
|  |  |  | Sensitivity analysis | - | 1.00 (0.26, 3.81) | 1.000 |
| MG-ADL scores at month 3 after thymectomy | 1.64 (0.44, 2.84) | 1.94 (0.57, 3.32) | Primary comparison | -0.30 (-2.02, 1.42) |  | 0.652 |
|  |  |  | Sensitivity analysis | -0.35 (-1.74, 1.04) |  | 0.616 |
| QMG scores at month 6 after thymectomy | 6.39 (4.79, 8.00) | 5.54 (3.95, 7.14) | Primary comparison | 0.85 (-1.76, 3.46) |  | 0.417 |
|  |  |  | Sensitivity analysis | 0.39 (-1.62, 2.40) |  | 0.697 |
| MG-ADL scores at month 6 after thymectomy | 1.14 (0.09, 2.19) | 0.99 (0.00, 1.97) | Primary comparison | 0.15 (-0.92, 1.22) |  | 0.710 |
|  |  |  | Sensitivity analysis | -0.04 (-0.97, 0.88) |  | 0.925 |
| Pred dose change (mg/d) | 0.77 (-2.52, 4.07) | 1.61 (-1.20, 4.41) | Primary comparison | -0.83 (-10.53, 8.86) |  | 0.823 |
|  |  |  | Sensitivity analysis | 1.63 (-6.56, 9.82) |  | 0.690 |
| Surgery outcomes |  |  |  |  |  |  |
| POMC | 13.7% (5.7%, 29.3%) | 15.3% (6.2%, 32.9%) | Primary comparison | - | 0.88 (0.10, 7.67) | 0.875 |
|  |  |  | Sensitivity analysis | - | 1.00 (0.17, 5.98) | 1.000 |
| Operative time (min) | 124.26 (117.18, 131.35) | 105.45 (100.47, 110.42) | Primary comparison | 18.82 (-21.64, 59.27) |  | 0.266 |
|  |  |  | Sensitivity analysis | 26.04 (-5.30, 57.39) |  | 0.101 |
| Blood loss (mL) | 88.52 (78.64, 98.39) | 57.46 (49.38, 65.53) | Primary comparison | 31.06 (-53.69, 115.80) |  | 0.366 |
|  |  |  | Sensitivity analysis | 3.91 (-41.03, 48.86) |  | 0.862 |
| ICU hours | 21.76 (16.49, 27.02) | 22.57 (17.45, 27.70) | Primary comparison | -0.82 (-10.53, 8.86) |  | 0.938 |
|  |  |  | Sensitivity analysis | 5.30 (-18.83, 29.43) |  | 0.660 |
| Assisted breathing time (hours) | 11.52 (7.95, 15.09) | 12.64 (8.46, 16.82) | Primary comparison | -1.13 (-16.76, 14.51) |  | 0.851 |
|  |  |  | Sensitivity analysis | -0.24 (-13.01, 12.53) |  | 0.970 |
| Pulmonary infection | 22.0% (11.2%, 38.7%) | 14.2% (5.6%, 31.6%) | Primary comparison | - | 1.70 (0.22, 13.10) | 0.486 |
|  |  |  | Sensitivity analysis | - | 2.35 (0.54, 12.51) | 0.273 |
| Postoperative hospitalization days | 5.70 (4.14, 7.26) | 6.77 (4.78, 8.76) | Primary comparison | -1.07 (-4.43, 2.29) |  | 0.427 |
|  |  |  | Sensitivity analysis | -0.44 (-3.12, 2.25) |  | 0.746 |
| Financial outcome |  |  |  |  |  |  |
| Surgery cost (CNY) | 38763.28 (38650.59, 38875.97) | 40602.15 (40485.55, 40718.75) | Primary comparison | -1838.87 (-15284.69, 11606.96) |  | 0.723 |
|  |  |  | Sensitivity analysis | 2727.08 (-8339.28, 13793.45) |  | 0.622 |

IS, immunosuppressants; MG-ADL, Activities of Daily Living of myasthenia gravis; POMC, postoperative myasthenic crisis; Pred, prednisone; QMG, Quantitative Myasthenia Gravis; RACT, rapid antibody clearance therapy.

Primary comparison: balancing Oral IS and RACT groups with overlap weighting; sensitivity analysis: balancing Oral IS and RACT groups with propensity score matching.

Notes: Costs are reported in Chinese Yuan. Surgical costs are presented as un-reimbursed (original) values, as the reimbursement rate for surgical expenses was identical across all groups, resulting in no between-group differences in net surgical costs after reimbursement.

Table S2. Generalized estimating equations (GEE) analysis of QMG and MG-ADL scores for models of RACT vs. Oral IS and models of EFG vs. LPE.

|  | QMG scores | | MG-ADL scores | |
| --- | --- | --- | --- | --- |
| **Model analysis** | β (95%CI) | p | β (95%CI) | p |
| **Main Effects& Interactions**  **(RACT-Oral IS)** |  |  |  |  |
| Intercept | 8.59 (7.76, 9.41) | <0.001 | 2.62 (1.89, 3.34) | <0.001 |
| Group effect | 0.00 (-1.23, 1.23) | 1.000 | -0.57 (-1.44, 0.30) | 0.199 |
| Time effect (Month3 – At surgery) | -1.05 (-2.81, 0.71) | 0.243 | -0.67 (-1.87, 0.53) | 0.274 |
| Time effect (Month6 – At surgery) | -3.04 (-4.57, -1.51) | <0.001 | -1.63 (-2.51, -0.75) | <0.001 |
| Interaction (Group*Month 3) | -0.23 (-2.53, 2.08) | 0.847 | 0.27 (-1.22, 1.76) | 0.722 |
| Interaction (Group*Month 6) | 0.85 (-1.36, 3.06) | 0.451 | 0.73 (-0.43, 1.88) | 0.217 |
| **Main Effects& Interactions**  **(EFG-LPE)** |  |  |  |  |
| Intercept | 15.62 (14.02, 17.21) | <0.001 | 6.91 (5.33, 8.50) | <0.001 |
| Group effect | 0.00 (-2.87, 2.87) | 1.000 | 0.04 (-2.68, 2.75) | 0.978 |
| Time effect (At surgery-Before RACT | -5.86 (-8.17, -3.55) | <0.001 | -4.65 (-6.36, -2.95) | <0.001 |
| Time effect (Month3 -Before RACT) | -8.35 (-11.21, 5.48) | <0.001 | -5.21 (-7.31, 3.11) | <0.001 |
| Time effect (Month6 – Before RACT) | -10.68 (-12.91, -8.46) | <0.001 | -6.34 (-7.98, -4.71) | <0.001 |
| Interaction (Group*At surgery) | -0.87 (-4.66, 2.92) | 0.654 | 0.10 (-2.90, 3.09) | 0.950 |
| Interaction (Group*Month 3) | 1.15 (-3.43, 5.73) | 0.623 | 0.77 (-2.73, 4.28) | 0.665 |
| Interaction (Group*Month 6) | 2.08 (-2.34, 6.49) | 0.357 | 1.16 (-1.81, 4.14) | 0.444 |

EFG, Efgartigimod; IS, immunosuppressants; LPE, lymphoplasmapheresis; MG-ADL, Activities of Daily Living of myasthenia gravis; QMG, Quantitative Myasthenia Gravis; RACT, rapid antibody clearance therapy.

Table S3. Factors associated with QMG scores at month 3 after thymectomy.

| **Characteristics** | **B (95%CI)** | **p** |
| --- | --- | --- |
| **Group**  Oral IS  PE  EFG | Reference  0.67 (-1.65, 2.99)  1.23 (-1.39, 3.85) | Reference  0.566  0.354 |
| **Sex**  Female  Male | Reference  -0.14 (-2.16, 1.88) | Reference  0.889 |
| BMI (kg/m^2^) | 0.02 (-0.26, 0.31) | 0.864 |
| Age of onset (y) | 0.01 (-0.08, 0.09) | 0.871 |
| MG Duration (months) | 0.04 (-0.01, 0.10) | 0.142 |
| Previous Crisis | 3.21 (-3.13, 9.55) | 0.316 |
| AChR-Ab positive | -0.66 (-9.38, 8.06) | 0.881 |
| Titin-Ab positive | -0.76 (-3.29, 1.77) | 0.549 |
| RyR-Ab positive | 1.37 (-1.49, 4.23) | 0.341 |
| Previous Pred | -0.85 (-3.02, 1.33) | 0.441 |
| Previous TAC | 0.31 (-1.70, 2.33) | 0.757 |
| Previous MMF | -1.13 (-4.90, 2.65) | 0.555 |
| Previous AZA | 0.13 (-6.25, 6.51) | 0.967 |
| TAC at surgery | -0.59 (-2.61, 1.43) | 0.564 |
| MMF at surgery | -0.61 (-4.73, 3.50) | 0.768 |
| Pred dose at surgery (mg/d) | -0.05 (-0.14, 0.04) | 0.277 |
| Hypertension | 0.78 (-1.78, 3.33) | 0.546 |
| Diabetes | 1.41 (-1.59, 4.41) | 0.353 |
| Autoimmunity | 0.56 (-2.76, 3.88) | 0.738 |
| Chronic respiratory disorders | -0.27 (-2.66, 2.13) | 0.825 |
| **Pathology**  Cyst  Hyperplasia  Thymoma  Undefined | Reference  -3.00 (-12.40, 6.40)  -0.32 (-9.25, 8.60)  -3.00 (-15.53, 9.53) | Reference  0.527  0.943  0.635 |
| **Thymoma WHO type**  A+AB+B1  B2+B3  Others  Non-thymoma | Reference  1.31 (-1.34, 3.95)  -1.21 (-7.86, 5.44)  -1.41 (-5.06, 2.23) | Reference  0.330  0.717  0.442 |
| **Surgery**  OPEN  RATS  VATS | Reference  -0.49 (-4.91, 3.93)  0.16 (-3.68, 4.00) | Reference  0.827  0.935 |
| Radiation | 0.33 (-1.70, 2.36) | 0.746 |
| QMG scores at surgery | 0.56 (0.25, 0.88) | <0.001 |

Ab, antibody; AChR, acetylcholine receptor; AZA, Azathioprine; BMI, body mass index; EFG, Efgartigimod; IS, immunosuppressants; LPE, lymphoplasmapheresis; MG, myasthenia gravis; MMF, Mycophenolate mofetil; pred, prednisone; OPEN, open thoracotomy; Pred, prednisone; QMG, Quantitative Myasthenia Gravis; RATS, robot-assisted thoracoscopic surgery; RACT, rapid antibody clearance therapy; RyR, Ryanodine Receptor; TAC, tacrolimus; VATS, video-assisted thoracoscopic surgery.

Table S4. Factors associated with POMC.

|  | **Univariable analysis** | | | **Multivariable analysis** | | |
| --- | --- | --- | --- | --- | --- | --- |
| **Characteristic** | **B (SE)** | **OR (95%CI)** | **p** | **B (SE)** | **OR (95%CI)** | **p** |
| Group  Oral IS  PE  EFG | Reference   - 1. (0.78)   -0.38 (1.19) | Reference  2.75 (0.61, 14.60)  0.69 (0.03, 5.86) | Ref  0.197  0.754 |  |  |  |
| Sex  Female  Male | Reference  1.52 (0.84) | Reference  4.55 (1.01, 32.05) | 0.071 | Reference  1.80 (1.22) | Reference  6.06 (0.72, 106.26) | 0.138 |
| BMI (kg/m^2^) | 0.30 (0.10) | 1.36 (1.13, 1.70) | 0.003 | 0.32 (0.12) | 1.38 (1.12, 1.83) | 0.007 |
| Age of onset (y) | -0.02 (0.03) | 0.98 (0.92, 1.04) | 0.424 |  |  |  |
| MG Duration (months) | 0.01 (0.02) | 1.01 (0.98, 1.04) | 0.363 |  |  |  |
| Previous Crisis | -14.56 (1696.73) | NA | 0.993 |  |  |  |
| AChR-Ab positive | 14.37 (2399.55) | NA | 0.995 |  |  |  |
| Titin-Ab positive | -17.66 (2776.67) | NA | 0.995 |  |  |  |
| RyR-Ab positive | -0.16 (1.13) | 0.85 (0.04, 5.78) | 0.886 |  |  |  |
| Previous Pred | 1.39 (1.09) | 4.00 (0.67, 76.51) | 0.204 |  |  |  |
| Previous TAC | -1.46 (0.84) | 0.23 (0.03, 1.05) | 0.082 |  |  |  |
| Previous MMF | 0.47 (1.16) | 1.60 (0.08, 11.74) | 0.685 |  |  |  |
| Previous AZA | -14.56 (1696.73) | NA | 0.993 |  |  |  |
| TAC at surgery | -2.11 (1.09) | 0.12 (0.01, 0.71) | 0.053 | -2.62 (1.50) | 0.07 (0.00, 0.86) | 0.080 |
| MMF at surgery | 0.71 (1.18) | 2.03 (0.10, 16.05) | 0.548 |  |  |  |
| Pred dose at surgery (mg/d) | 0.04 (0.03) | 1.04 (0.97, 1.11) | 0.311 |  |  |  |
| Hypertension | 0.87 (0.78) | 2.38 (0.45, 10.43) | 0.265 |  |  |  |
| Diabetes | -0.18 (1.12) | 0.83 (0.04, 5.39) | 0.871 |  |  |  |
| Autoimmunity | 0.10 (1.13) | 1.11 (0.06, 7.49) | 0.928 |  |  |  |
| Chronic respiratory disorders | 1.68 (0.74) | 5.39 (1.26, 24.56) | 0.023 |  |  |  |
| Pathology  Cyst  Hyperplasia  Thymoma  Undefined | Reference  0 (6918.30)  16.69 (6522.64)  0 (9224.40) | Reference  NA  NA  NA | Reference  1.000  0.998  1.000 |  |  |  |
| Thymoma WHO type, n (%)  A+AB+B1  B2+B3  Others  Non-thymoma | Reference  0.86 (1.11)  -16.00 (4612.20)  -16.00 (2062.64) | Reference  2.36 (0.38, 45.93)  NA  NA | Reference  0.437  0.997  0.994 |  |  |  |
| Surgery, n (%)  OPEN  RATS  VATS | Reference  -1.79 (1.35)  -1.49 (0.97) | Reference  0.17 (0.01, 2.19)  0.23 (0.04, 1.87) | Reference  0.186  0.125 |  |  |  |
| Radiation, n (%) | 1.08 (0.75) | 2.93 (0.71, 14.80) | 0.151 |  |  |  |
| QMG scores at surgery | 0.30 (0.12) | 1.35 (1.07, 1.74) | 0.014 | 0.47 (0.19) | 1.59 (1.15, 2.50) | 0.013 |

Ab, antibody; AChR, acetylcholine receptor; AZA, Azathioprine; BMI, body mass index; EFG, Efgartigimod; IS, immunosuppressants; LPE, lymphoplasmapheresis; MG, myasthenia gravis; MMF, Mycophenolate mofetil; OPEN, open thoracotomy; Pred, prednisone; QMG, Quantitative Myasthenia Gravis; RATS, robot-assisted thoracoscopic surgery; RACT, rapid antibody clearance therapy; RyR, Ryanodine Receptor; TAC, tacrolimus; VATS, video-assisted thoracoscopic surgery.

† Extreme odds ratios with non-informative confidence intervals (e.g., OR > 1×10⁵ or CI width > 1×10¹⁰⁰) resulting from quasi-complete separation in the univariable models are reported as NA.

Table S5 Baseline characteristics of patients with RACT vs. Oral IS within 3 months before thymectomy before and after propensity score matching.

| **Variable** | **Levels** | **Crude (Unmatched)** | | | | **After Matching** | | | |
| --- | --- | --- | --- | --- | --- | --- | --- | --- | --- |
|  |  | **Oral IS** | **RACT** | **p** | **SMD** | **Oral IS** | **RACT** | **p** | **SMD** |
| n |  | 36 | 42 |  |  | 23 | 23 |  |  |
| Sex, n (%) | Female  Male | 22 (61.1%)  14 (38.9%) | 19 (45.2%)  23 (54.8%) | 0.162 | 0.322 | 14 (60.9%)  9 (39.1%) | 12 (52.2%)  11 (47.8%) | 0.552 | 0.176 |
| BMI (kg/m^2^) |  | 23.53 [22.38, 25.15] | 23.21 [20.93, 26.03] | 0.548 | 0.045 | 24.03 [22.66, 25.51] | 22.49 [20.60, 24.74] | 0.081 | 0.419 |
| Age of onset (y) |  | 46.8±11.4 | 48.9±12.1 | 0.435 | 0.179 | 47.1±10.8 | 47.59±10.3 | 0.881 | 0.044 |
| MG Duration (months) |  | 4.50 [3.00, 11.00] | 4.00 [2.00, 8.00] | 0.389 | 0.217 | 4.00 [3.00, 8.00] | 3.00 [2.00, 4.50] | 0.175 | 0.171 |
| Previous Crisis, n (%) |  | 1 (2.8%) | 1 (2.4%) | 1.000 | 0.025 | 1 (4.3%) | 1 (4.3%) | 1.000 | <0.001 |
| AChR-Ab positive, n (%) |  | 29 (96.7%) | 41 (100.0%) | 0.423 | 0.263 | 19 (95.0%) | 22 (100.0%) | 0.476 | 0.324 |
| Titin-Ab positive, n (%) |  | 8 (27.6%) | 7 (17.5%) | 0.316 | 0.243 | 3 (15.0%) | 2 (9.5%) | 0.663 | 0.168 |
| RyR-Ab positive, n (%) |  | 4 (13.8%) | 7 (17.9%) | 0.747 | 0.114 | 3 (15.0%) | 2 (10.0%) | 1.000 | 0.152 |
| Previous Pred, n (%) |  | 30 (83.3%) | 24 (57.1%) | 0.012 | 0.598 | 17 (73.9%) | 16 (69.6%) | 0.743 | 0.097 |
| Previous TAC, n (%) |  | 18 (50.0%) | 22 (52.4%) | 0.834 | 0.048 | 13 (56.5%) | 10 (43.5%) | 0.376 | 0.263 |
| Previous MMF, n (%) |  | 2 (5.6%) | 4 (9.5%) | 0.681 | 0.151 | 0 (0.0%) | 2 (8.7%) | 0.489 | 0.436 |
| Previous AZA, n (%) |  | 2 (5.6%) | 0 (0.0%) | 0.210 | 0.343 | 0 (0.0%) | 0 (0.0%) | NA | <0.001 |
| Tac at surgery, n (%) |  | 17 (47.2%) | 19 (45.2%) | 0.861 | 0.040 | 13 (56.5%) | 8 (34.8%) | 0.139 | 0.447 |
| MMF at surgery, n (%) |  | 1 (2.8%) | 4 (9.5%) | 0.366 | 0.284 | 0 (0.0%) | 2 (8.70%) | 0.489 | 0.436 |
| Pred dose at surgery (mg/d) |  | 20.00 [3.75, 20.00] | 20.00 [0.00, 23.75] | 0.692 | 0.138 | 20.00 [0.00, 20.00] | 20.00 [0.00, 22.50] | 0.290 | 0.245 |
| Hypertension, n (%) |  | 8 (22.2%) | 7 (16.7%) | 0.535 | 0.141 | 6 (26.1%) | 2 (8.7%) | 0.277 | 0.436 |
| Diabetes, n (%) |  | 6 (16.7%) | 4 (9.5%) | 0.500 | 0.213 | 5 (21.7%) | 3 (13.0%) | 0.699 | 0.231 |
| Autoimmunity, n (%) |  | 4 (11.1%) | 4 (9.5%) | 1.000 | 0.052 | 1 (4.3%) | 3 (13.0%) | 0.608 | 0.312 |
| Chronic respiratory disorders, n (%) |  | 5 (13.9%) | 13 (31.0%) | 0.075 | 0.418 | 4 (17.4%) | 3 (13.0%) | 1.000 | 0.121 |
| Pathology, n (%) | Cyst  Hyperplasia  Thymoma  Undefined | 0 (0.0%)  7 (19.4%)  28 (77.8%)  1 (2.8%) | 1 (2.4%)  1 (2.4%)  40 (95.2%)  0 (0.0%) | 0.012 | 0.671 | 0 (0.0%)  2 (8.7%)  21 (91.3%) | 0 (0.0%)  1 (4.3%)  22 (95.7%) | 1.000 | 0.177 |
| Thymoma WHO type, n (%) | A+AB+B1  B2+B3  Others  Non-thymoma | 6 (16.7%)  21 (58.3%)  1 (2.8%)  8 (22.2%) | 8 (19.0%)  31 (73.8%)  1 (2.4%)  2 (4.8%) | 0.105 | 0.534 | 5 (21.7%)  15 (65.2%)  1 (4.3%)  2 (8.7%) | 5 (21.7%)  16 (69.6%)  1 (4.3%)  1 (4.3%) | 1.000 | 0.179 |
| Surgery, n (%) | OPEN  RATS  VATS | 2 (5.6%)  8 (22.2%)  26 (72.2%) | 4 (9.5%)  5 (11.9%)  33 (78.6%) | 0.433 | 0.301 | 1 (4.3%)  3 (13.0%)  19 (82.6%) | 3 (13.0%)  3 (13.0%)  17 (73.9%) | 0.683 | 0.315 |
| Radiation, n (%) |  | 18 (50.0%) | 16 (38.1%) | 0.291 | 0.242 | 11 (47.8%) | 11 (47.8%) | 1.000 | <0.001 |
| QMG scores before thymectomy |  | 7.92±2.79 | 9.12±3.09 | 0.078 | 0.408 | 8.00 [8.00, 10.00] | 9.00 [8.00, 9.50] | 0.883 | 0.035 |
| MG-ADL scores before thymectomy |  | 1.50 [0.00, 4.00] | 2.00 [1.00, 3.00] | 0.639 | 0.038 | 2.61±1.83 | 1.83±1.23 | 0.096 | 0.502 |

Ab, antibody; AChR, acetylcholine receptor; AZA, Azathioprine; BMI, body mass index; ab, antibody; IS, immunosuppressants; MG-ADL, Activities of Daily Living of myasthenia gravis; MMF, Mycophenolate mofetil; pred, prednisone; OPEN, open thoracotomy; Pred, prednisone; QMG, Quantitative Myasthenia Gravis; RATS, robot-assisted thoracoscopic surgery; RACT, rapid antibody clearance therapy; RyR, Ryanodine Receptor; SMD, standardized mean differences; TAC, tacrolimus; VATS, video-assisted thoracoscopic surgery.

Table S6. Baseline characteristics of patients with EFG vs. LPE within 3 months before thymectomy before and after propensity score matching.

| **Variable** | **level** | **Crude (Unmatched)** | | | | **After Matching** | | | |
| --- | --- | --- | --- | --- | --- | --- | --- | --- | --- |
|  |  | **LPE** | **EFG** | **p** | **SMD** | **LPE** | **EFG** | **p** | **SMD** |
| n |  | 16 | 17 |  |  | 10 | 10 |  |  |
| Sex, n (%) | Female | 9 (56.2%) | 8 (47.1%) | 0.598 | 0.185 | 6 (60.0%) | 4 (40.0%) | 0.371 | 0.408 |
|  | Male | 7 (43.8%) | 9 (52.9%) |  |  | 4 (40.0%) | 6 (60.0%) |  |  |
| BMI (kg/m^2^) |  | 23.76 [20.98, 26.80] | 23.18 [21.11, 24.14] | 0.540 | 0.317 | 23.76 [21.40, 26.79] | 23.21 [21.64, 24.58] | 0.850 | 0.109 |
| Age of onset (y) |  | 45.8±10.2 | 51.1±13.7 | 0.218 | 0.440 | 41.5 [34.0, 47.3] | 51.7 [34.2, 56.5] | 0.623 | 0.259 |
| MG duration (months) |  | 3.50 [2.00, 6.50] | 4.00 [2.00, 7.00] | 0.956 | 0.260 | 3.50 [2.25, 7.00] | 3.50 [1.25, 6.75] | 0.593 | 0.241 |
| Previous crisis, n (%) |  | 1 (6.2%) | 0 (0.0%) | 0.485 | 0.365 | 0 (0.0%) | 0 (0.0%) | NA | <0.001 |
| AChR-Ab, n (%) |  | 15 (100.0%) | 17 (100.0%) | NA | <0.001 | 9 (100.0%) | 10 (100.0%) | NA | <0.001 |
| Titin-Ab, n (%) |  | 1 (6.7%) | 5 (29.4%) | 0.178 | 0.619 | 1 (11.1%) | 4 (40.0%) | 0.303 | 0.702 |
| RyR-Ab, n (%) |  | 1 (7.1%) | 4 (23.5%) | 0.344 | 0.467 | 1 (11.1%) | 2 (20.0%) | 1.000 | 0.247 |
| Previous pred, n (%) |  | 11 (68.8%) | 6 (35.3%) | 0.055 | 0.711 | 5 (50.0%) | 6 (60.0%) | 1.000 | <0.001 |
| Previous tac, n (%) |  | 9 (56.2%) | 9 (52.9%) | 0.849 | 0.066 | 7 (70.0%) | 5 (50.0%) | 0.650 | 0.417 |
| Previous MMF, n (%) |  | 1 (6.2%) | 2 (11.8%) | 1.000 | 0.194 | 0 (0.0%) | 1 (10.0%) | 1.000 | 0.471 |
| Previous AZA, n (%) |  | 0 (0.0%) | 0 (0.0%) | NA | <0.001 | 0 (0.0%) | 0 (0.0%) | NA | <0.001 |
| TAC at surgery, n (%) |  | 7 (43.8%) | 8 (47.1%) | 0.849 | 0.066 | 6 (60.0%) | 4 (40.0%) | 0.371 | 0.408 |
| MMF at surgery, n (%) |  | 1 (6.2%) | 2 (11.8%) | 1.000 | 0.194 | 0 (0.0%) | 1 (10.0%) | 1.000 | 0.471 |
| Pred dose at surgery (mg/d) |  | 20.00 [0.00, 25.00] | 0.00 [0.00, 20.00] | 0.095 | 0.555 | 20.00 [0.00, 25.00] | 15.00 [0.00, 20.00] | 0.665 | 0.156 |
| Hypertension, n (%) |  | 2 (12.5%) | 2 (11.8%) | 1.000 | 0.023 | 1 (10.0%) | 2 (20.0%) | 1.000 | 0.283 |
| Diabetes, n (%) |  | 0 (0.0%) | 1 (5.9%) | 1.000 | 0.354 | 0 (0.0%) | 0 (0.0%) | NA | <0.001 |
| Autoimmunity, n (%) |  | 4 (25.0%) | 0 (0.0%) | 0.044 | 0.816 | 0 (0.0%) | 0 (0.0%) | NA | <0.001 |
| Chronic respiratory disorder, n (%) |  | 6 (37.5%) | 5 (29.4%) | 0.622 | 0.172 | 4 (40.0%) | 4 (40.0%) | 1.000 | <0.001 |
| Pathology, n (%) | Non-thymoma  Thymoma | 1 (6.2%)  15 (93.8%) | 1 (5.9%)  16 (94.1%) | 1.000 | 0.015 | 1 (10.0%)  9 (90.0%) | 0 (0.0%)  10 (100.0%) | 1.000 | 0.471 |
| Thymoma WHO type, n (%) | A+AB+B1  B2+B3  Others | 5 (31.2%)  9 (56.2%)  2 (12.5%) | 2 (11.8%)  14 (82.4%)  1 (5.9%) | 0.328 | 0.592 | 3 (30.0%)  6 (60.0%)  1 (10.0%) | 2 (20.0%)  8 (80.0%)  0 (0.0%) | 0.624 | 0.567 |
| Radiation, n (%) |  | 7 (43.8%) | 3 (17.6%) | 0.141 | 0.590 | 5 (50.0%) | 2 (20.0%) | 0.350 | 0.663 |
| QMG scores before RACT |  | 16.69±3.34 | 15.71±5.05 | 0.518 | 0.229 | 15.30±2.95 | 12.90±3.98 | 0.143 | 0.685 |
| MG-ADL scores before RACT |  | 7.44±2.56 | 7.24±4.82 | 0.881 | 0.052 | 6.90±2.88 | 4.80±3.12 | 0.136 | 0.699 |
| QMG scores before surgery |  | 9.00 [7.75; 10.00] | 8.00 [6.00; 11.00] | 0.276 | 0.325 | 9.00 [7.25, 10.00] | 9.00 [5.25, 11.75] | 0.648 | 0.239 |
| MG-ADL scores before surgery |  | 2.50 [2.00; 3.00] | 2.00 [1.00; 4.00] | 0.397 | 0.124 | 2.00 [1.25, 3.00] | 2.00 [2.00, 4.00] | 0.414 | 0.570 |

Ab, antibody; AChR, acetylcholine receptor; AZA, Azathioprine; BMI, body mass index; EFG, Efgartigimod; LPE, lymphoplasmapheresis; MG-ADL, Activities of Daily Living of myasthenia gravis; MMF, Mycophenolate mofetil; pred, prednisone; OPEN, open thoracotomy; Pred, prednisone; QMG, Quantitative Myasthenia Gravis; RATS, robot-assisted thoracoscopic surgery; RyR, Ryanodine Receptor; SMD, standardized mean differences; TAC, tacrolimus; VATS, video-assisted thoracoscopic surgery..

Table S7. E-value analyses for the EFG vs. LPE on outcomes.

| **Outcomes** | **Cohens’d (95%CI)** | **Treatment effect E-Value (Point Estimate)** | **Treatment effect E-Value (Conservative Estimate)** | **Strongest baseline variable** | **E-Value of Baseline Variable** | **P-Value of Baseline Variable** |
| --- | --- | --- | --- | --- | --- | --- |
| Blood loss (mL) | -0.57 (-1.70, 0.57) | 1.72 | 1.72 | Previous crisis | 3.84 | <0.001 |
| Operative time (min) | -0.76 (-1.93, 0.40) | 2.02 | 1.48 | Previous crisis | 4.40 | <0.001 |
| Preoperative costs (original) (CNY) | 1.16 (0.23, 2.09) | 2.69 | 1.25 | Previous MMF use | 4.69 | 0.098 |
| Surgery costs (CNY) | -0.87 (-1.98, 0.25) | 2.19 | 1.28 | RyR-Ab | 2.04 | 0.070 |
| Postoperative costs (original) (CNY) | 0.73 (-0.29, 1.75) | 1.96 | 1.33 | RyR-Ab | 2.23 | 0.065 |
| Perioperative costs (original) (CNY) | 0.83 (-0.23, 1.88) | 2.12 | 1.25 | TAC | 1.90 | 0.120 |

CNY, Chinese Yuan; EFG, efgartigimod; MG-ADL, Activities of Daily Living of myasthenia gravis.

**E-value Analysis**

For treatment effects that were marginally significant, we conducted E-value analyses to assess their robustness to unmeasured confounding. The E-value represents the minimum strength of association, on the risk ratio scale, that an unmeasured confounder would need to have with both the treatment assignment and the outcome to fully explain away the observed treatment effect.

We calculated standardized effect sizes (Cohen's d) by dividing the treatment effect estimate by the pooled standard deviation derived from the overlap-weighted samples. E-values were then computed using the formula: $Evalue=\sqrt{1+d^{2}}+|d|$, as described by VanderWeele and Ding. We reported both the point estimate E-value and a conservative E-value based on the confidence interval boundary closer to the null value (upper bound for negative effects, lower bound for positive effects).

To contextualize the magnitude of these E-values, we compared them with the strength of known baseline-outcome associations in the same weighted population. For each outcome, we identified the baseline variable with the largest E-value and compared it with the treatment effect E-values. This comparison assesses whether stronger unmeasured confounding would be required to explain away the treatment effect than to explain away associations with established risk factors.


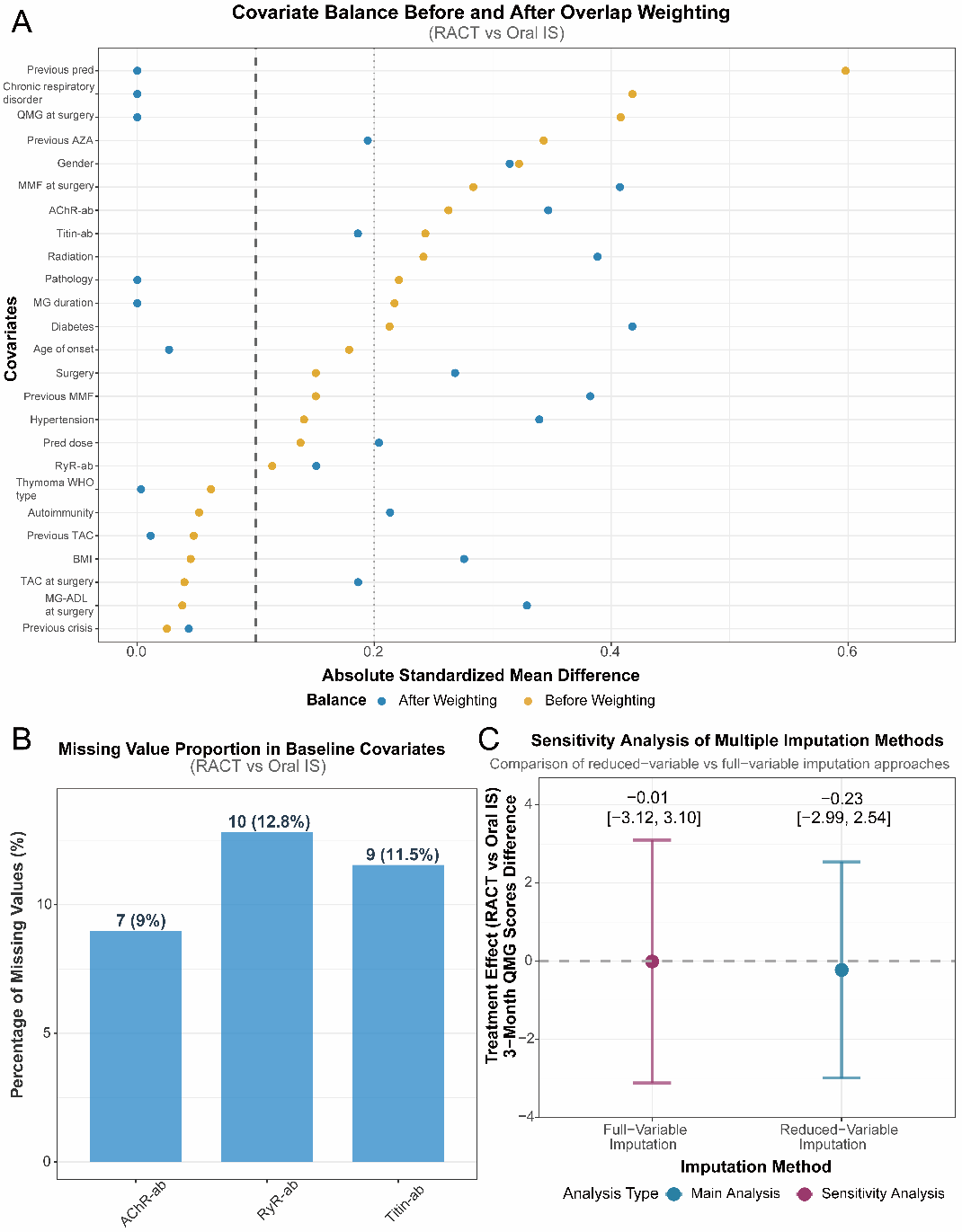


**Figure S1.** **Covariate balancing, missing data patterns, and sensitivity analysis for the Oral IS vs. RACT Comparison.** (A) Covariate balance before and after overlap weighting. Love plot displaying the absolute standardized mean differences (SMD) for all baseline covariates prior to (orange circles) and following (blue circles) the application of overlap weighting after adjusting five key variables: QMG scores at surgery, previous prednisone use, thymic pathology, chronic respiratory disorder, and MG duration. (B) Distribution of Missing baseline data. (C) Sensitivity analysis of multiple imputation strategies. Comparison of the primary analysis model (using multiple imputation for missing antibody data, based on five key clinically relevant variables) against a sensitivity analysis model (which leveraged the full set of all available baseline covariates for imputation). The nearly identical point estimates and substantially overlapping confidence intervals for the treatment effect on the 3-month QMG score (Primary: -0.23, 95% CI: -2.99 to 2.54; Sensitivity: -0.01, 95% CI: -3.12 to 3.10) demonstrate that the findings from the more parsimonious primary model are robust.


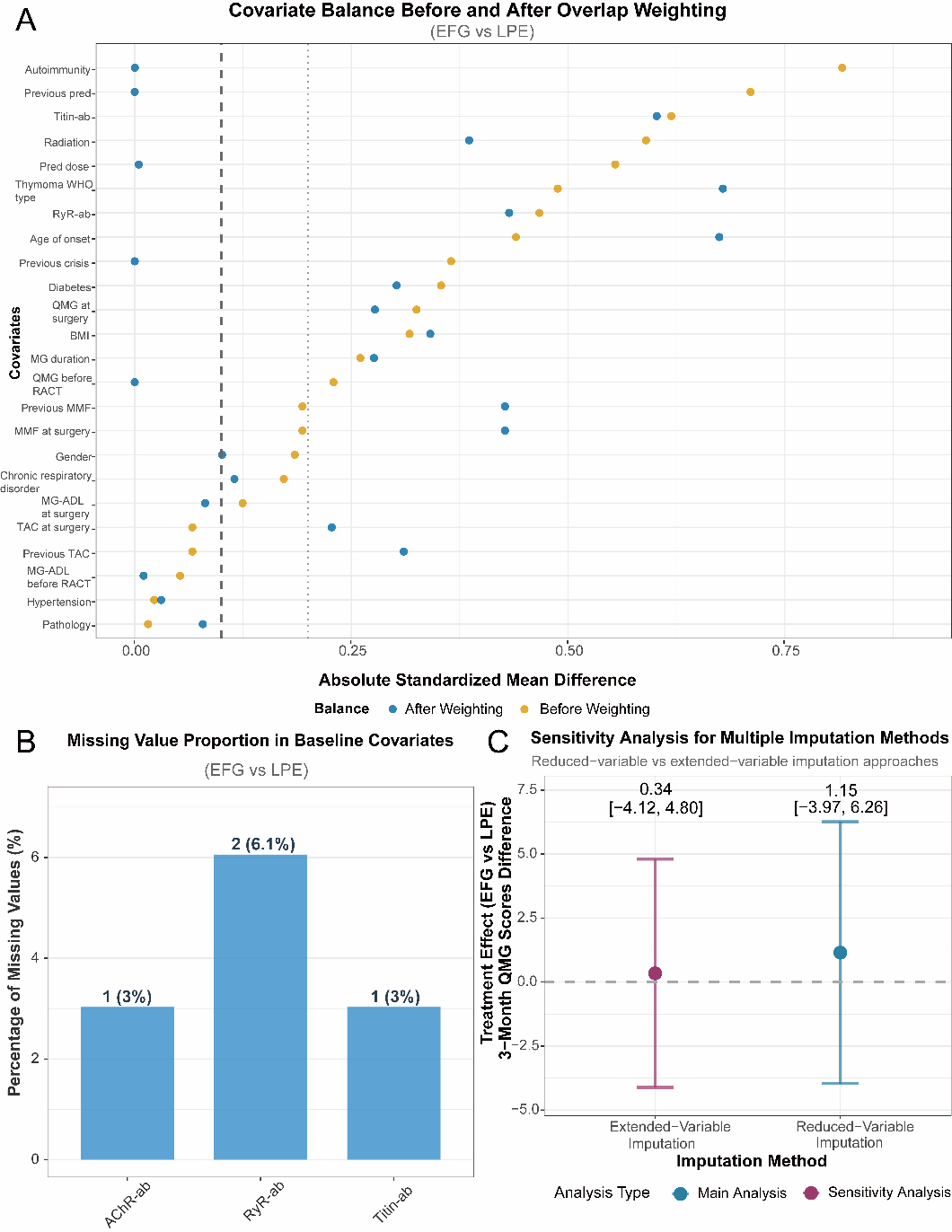


**Figure S2. Covariate balancing, missing data patterns, and sensitivity analysis for the LPE vs. EFG Comparison**. (A) Covariate balance before and after overlap weighting. Love plot displaying the absolute standardized mean differences (SMD) for all baseline covariates prior to (orange circles) and following (blue circles) the application of overlap weighting after adjusting three key variables: QMG scores before RACT, previous prednisone use, autoimmunity. (B) Distribution of Missing baseline data. (C) Sensitivity analysis of multiple imputation strategies. Comparison of the primary analysis model (using multiple imputation for missing antibody data, based on three key clinically relevant variables) against a sensitivity analysis model (which leveraged another two variables: MG duration and pathology for imputation). The nearly identical point estimates and substantially overlapping confidence intervals for the treatment effect on the 3-month QMG score (Primary: 1.15, 95%CI: -3.97 to 6.26; Sensitivity: 0.34, 95%CI: -4.12 to 4.80) demonstrate that the findings from the more parsimonious primary model are robust.


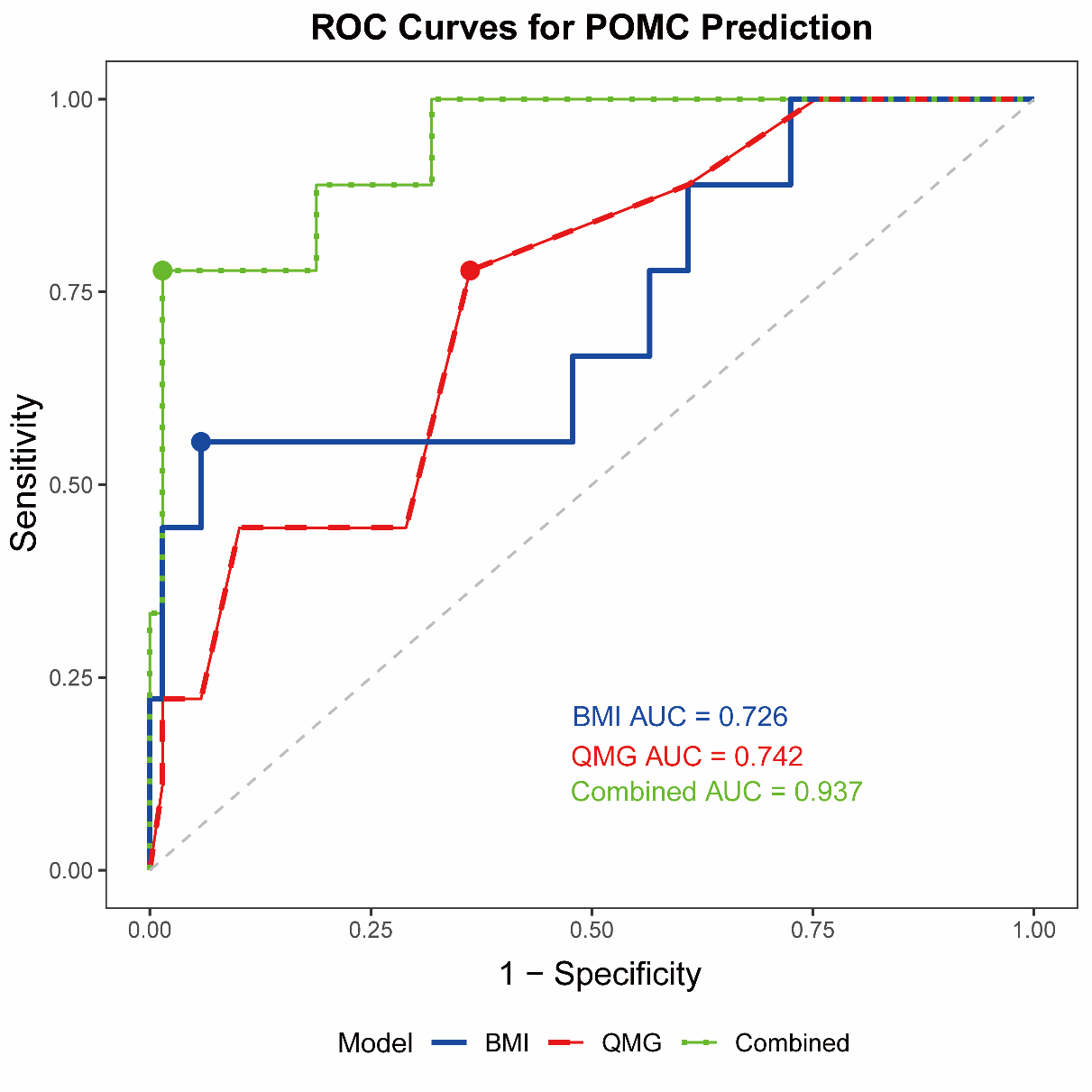


**Figure S3. ROC curves for POMC prediction.** Green line: combined model (BMI + QMG + TAC at surgery + Sex). The optimal cut-off point is 0.40 (AUC = 0.937, sensitivity = 0.778, specificity = 0.986). Red line: QMG model. The optimal cut-off point is 8.5 (AUC=0.742, sensitivity=0.778, specificity=0.638). Blue line: BMI model. The optimal cut-off point is 27.99 kg/m^2^ (AUC=0.726, sensitivity=0.556, specificity=0.942).
